# PhyberSIM: a tool for the generation of ground truth to evaluate brain fiber clustering algorithms

**DOI:** 10.3389/fnins.2024.1396518

**Published:** 2024-05-30

**Authors:** Elida Poo, Jean-François Mangin, Cyril Poupon, Cecilia Hernández, Pamela Guevara

**Affiliations:** ^1^Department of Electrical Engineering, Faculty of Engineering, Universidad de Concepción, Concepción, Chile; ^2^CEA, CNRS, Baobab, Neurospin, Université Paris-Saclay, Gif-sur-Yvette, France; ^3^Department of Computer Science, Faculty of Engineering, Universidad de Concepción, Concepción, Chile; ^4^Center for Biotechnology and Bioengineering (CeBiB), Santiago, Chile

**Keywords:** fiber clustering, tractography, fiber bundle simulator, spline curves, whole-brain datasets

## Abstract

Diffusion Magnetic Resonance Imaging tractography is a non-invasive technique that produces a collection of streamlines representing the main white matter bundle trajectories. Methods, such as fiber clustering algorithms, are important in computational neuroscience and have been the basis of several white matter analysis methods and studies. Nevertheless, these clustering methods face the challenge of the absence of ground truth of white matter fibers, making their evaluation difficult. As an alternative solution, we present an innovative brain fiber bundle simulator that uses spline curves for fiber representation. The methodology uses a tubular model for the bundle simulation based on a bundle centroid and five radii along the bundle. The algorithm was tested by simulating 28 Deep White Matter atlas bundles, leading to low inter-bundle distances and high intersection percentages between the original and simulated bundles. To prove the utility of the simulator, we created three whole-brain datasets containing different numbers of fiber bundles to assess the quality performance of QuickBundles and Fast Fiber Clustering algorithms using five clustering metrics. Our results indicate that QuickBundles tends to split less and Fast Fiber Clustering tends to merge less, which is consistent with their expected behavior. The performance of both algorithms decreases when the number of bundles is increased due to higher bundle crossings. Additionally, the two algorithms exhibit robust behavior with input data permutation. To our knowledge, this is the first whole-brain fiber bundle simulator capable of assessing fiber clustering algorithms with realistic data.

## 1 Introduction

Diffusion Magnetic Resonance Imaging (dMRI) is a non-invasive technique that measures the random motion of water molecules in tissues (Le Bihan and Iima, 2015). Fiber tractography based on dMRI reconstructs the main fiber pathways of brain white matter (WM), offering enormous potential for the study of structural brain connectivity along life span and different conditions (Dennis et al., 2013; Mimenza et al., 2020; Zhang et al., 2022). The tractography data are complex due to their large number of fibers, their high proportion of noisy fibers, and the complex morphology of brain connections, which presents significant challenges for their analysis (Maier-Hein et al., 2017; Yang et al., 2021).

There are several methods to analyze tractography data, including fiber clustering algorithms, which have made an important contribution due to their ability to identify similar fibers automatically and discard noisy fibers (O'Donnell et al., 2006; Guevara et al., 2011b; Garyfallidis et al., 2012; Reichenbach et al., 2015; Huerta et al., 2020; Vázquez et al., 2020; Legarreta et al., 2022). These methods analyze a collection of 3D tractography streamlines and group them into clusters or bundles that contain fibers with similar shapes and spatial positions. Although most exploratory clustering methods do not directly incorporate anatomical information, they are usually used as a step in a more extended processing pipeline with a specific goal, e.g., creating a WM bundle atlas, performing WM bundle segmentation, filtering WM fibers, or generating a diffusion-based parcellation.

Numerous fiber clustering techniques have been described in the literature. One of the most widely used methods in the state-of-the-art is QuickBundles (QB) (Garyfallidis et al., 2012). It measures the similarity between fibers based on a distance metric called the Minimum Average Direct Flip (MDF). The advantages of using the MDF distance are its speed in computation and the consideration of streamline directionality. Using the MDF distance requires that all fibers have the same number of points, so an initial step of the method interpolates the input fibers. By defining a distance threshold, the algorithm determines which streamlines belong to the same bundle or cluster without recomputing the clusters. QB has the benefit of being a simple and fast algorithm for identifying fiber bundles and reducing the dimensionality of large tractography datasets. This algorithm has been used in multiple applications, for example, WM bundle segmentation (Garyfallidis et al., 2018) and fiber filtering (Feng et al., 2023).

Another fiber clustering algorithm is Fast Fiber Clustering (FFClust) (Vázquez et al., 2020), which was designed mainly to identify compact clusters in massive tractography datasets with reduced computation time. This algorithm consists of four steps. It first reduces the dimensionality of the data by applying a MiniBatch K-Means (Sculley, 2010) on a subset of fiber points. The fibers whose points share the same point clusters are then grouped into preliminary fiber clusters. Then, it reassigns small groups of preliminary clusters to the nearest large clusters based on a maximum distance threshold. The last step uses a graph representation and a maximal clique algorithm to merge candidate clusters into final clusters. This algorithm has been used for the creation of a superficial WM bundle atlas (Román et al., 2022), a method for diffusion-based cortical parcellation (Molina et al., 2023), and outlier removal.

However, despite their usefulness, these algorithms are difficult to evaluate, compare, and improve due to the absence of ground truth. Different fiber clustering algorithms produce different fiber clusters from a tractography dataset. Furthermore, the same algorithm generates different clusters depending on the input parameters. Therefore, the development of robust evaluation strategies is imperative to provide users with reliable metrics in the clustering results. The existing literature offers limited tools for evaluating and comparing different fiber clustering results for whole-brain tractography datasets. In most cases, researchers employ alternative techniques and metrics to evaluate the performance of their clustering algorithms.

For example, the authors of QuickBundles used different whole-brain tractography datasets with initial conditions and four metrics to evaluate their algorithm. These metrics are the optimized matched agreement (OMA), coverage, overlap, and Bundle Adjacency (BA). The OMA was used to compare the different clusterings that arise when random permutations are applied to the input fiber dataset. Coverage and overlap metrics were calculated to evaluate how QB centroids are a better reduction than an equivalent number of random selections of streamlines. Finally, the BA was employed to compare the clustering results between subjects. The authors performed a simple fiber simulation that generated three distinct bundles of streamlines made from different combinations of sinusoidal and helicoidal functions, which illustrated the algorithm's behavior for low and high thresholds.

On the other hand, the authors of the FFClust algorithm (Vázquez et al., 2020) provided a performance evaluation with other state-of-the-art methods, such as a robust clustering proposal for intra-subject analysis (Guevara et al., 2011b), QuickBundles (Garyfallidis et al., 2012), and an improvement of QuickBundles called QuickBundlesX (Garyfallidis et al., 2016). The quality of the clusters obtained by each algorithm was evaluated using the intra-cluster and inter-cluster distances and the Davies–Bouldin (DB) index (Xu and Tian, 2015), defined as the average similarity between each cluster with its most similar cluster. In addition, they compared the execution times of the algorithms and found that their parallel implementation is about 8.6 times faster than QB using five threads.

Although the metrics and comparisons used have allowed a partial evaluation of the behavior of these algorithms, it would be desirable to have a simulation tool that allows validation with data that provide a ground truth at a whole-brain scale. However, the simulation of brain fibers is challenging due to their irregular and complex shape, making it difficult to create realistic data. Currently, most available simulation frameworks have been developed to validate tractography algorithms or local diffusion models.

Close et al. (2009) proposed an interesting tool that uses a collection of numerical constructs known as strands. This approach involves simulating bundles as coarse strands that are 3D linear splines with constant circular cross-sections. The simulation starts by initializing these strands along straight-line segments, connecting randomly generated points on the surface of a sphere until the entire sphere is covered. This tool has been proven helpful in validating fiber tracking algorithms by providing realistic bundle configurations for this purpose. Also, Neher et al. (2014) proposed an open-source framework called Fiberfox that allows for the intuitive definition of various fiber tract configurations, such as twisting, fanning, highly curved, kissing, and crossing. It was used to replicate the FiberCup physical phantom (Poupon et al., 2008) with different MRI artifacts and realistic microstructural parameters of white matter to compare different diffusion models and tractography algorithms.

Another idea is the analysis of fiber bundles using cross-sectional data to represent the bundle shapes. An example was proposed by Glozman et al. (2018), who developed a framework to evaluate age-dependent changes in bundle shape between subjects. To accomplish this, they used a geometric model to study the fascicle shapes, which divides a bundle into cross-sections and extracts a set of seed points, accurately capturing its shape.

Some fiber bundle simulators have been created to validate clustering algorithms. Guevara et al. (2011b) proposed a fiber fascicle simulation to validate their own fiber clustering algorithm. The simulated data were generated from a set of centroids, defined by randomly selected fibers from the whole-brain tractography dataset of a subject. The bundles were formed using a random translation of their centroids. The output was a set of cylindrical bundles across the whole brain, with a variable number of fibers added to simulate noise.

Following this idea, Poo et al. (2023) added exponential curves for a more realistic representation of the fiber bundle with dispersed ends. The simulator was validated by generating simulated bundles from a deep WM bundle atlas (Guevara et al., 2012). For this evaluation, to obtain more realistic simulations, the bundles were subdivided into five sub-bundles, each one created by the input simulation parameters: the centroid, the radii of each end, the central radius, the dispersion starting point for the exponential at each end, and the number of fibers. Furthermore, to show the applicability of this tool, four whole-brain fiber bundle datasets were created and used to evaluate the performance of a fiber clustering algorithm (QB) with 100 and 500 bundles and two end radii ranges (5–10 and 10–15 mm). These simulations used only one centroid per bundle.

This paper aims to create a more realistic simulation of brain fibers with intuitive and flexible parameters, to be used as a ground truth for evaluating different clustering algorithms. To achieve a realistic appearance, we used spline curves for fiber representation. A spline curve is defined by a set of control points and a set of mathematical functions that approximate the curve between these control points, enabling the representation of more complex shapes, such as brain fibers. These curves have previously been used to represent the fiber shape in fiber tracking algorithms (Wu et al., 2009, 2018; Losnegård et al., 2013).

For simulating a bundle, we propose a tubular model with variable radii, defined by a bundle centroid and the radii of five cross-sections across the bundle. Other parameters include the number of fibers and the mean and standard deviation of Gaussian noise at the bundle ends. To evaluate the simulator, we simulated bundles from a deep WM atlas (Guevara et al., 2012). We compared them with the original bundles, obtaining good similarity between the bundles, with a mean intersection percentage of 76.5%, which outperforms a previous, more straightforward, simulator based on exponential curves (Poo et al., 2023).

Furthermore, three simulated whole-brain datasets with different numbers of bundles were created to serve as ground truth for evaluating the performance of two clustering algorithms: QuickBundles (Garyfallidis et al., 2012) and FFClust (Vázquez et al., 2020). The evaluation was carried out by measuring five clustering metrics: Accuracy, Precision, Recall, F-measure and the Maximun matching ratio that provided insights into the quality of the clusters detected by the algorithms. Additionally, both algorithms were evaluated with permutations of the input data. The results show the applicability of the proposed simulator to perform an objective evaluation of the performance of fiber clustering algorithms with realistic data. Despite its limitations, to the best of our knowledge, this is the first whole-brain fiber bundle simulator capable of addressing the lack of ground truth for these types of algorithms. The code of the PhyberSIM is available at https://github.com/elidapoo/Brain_bundle_simulator.

## 2 Materials and methods

### 2.1 Materials

#### 2.1.1 Tractography datasets

We used the HARDI ARCHI database (Schmitt et al., 2012) to evaluate the simulator and extract random fibers to generate whole-brain tractography datasets. Data were acquired using special acquisition sequences on a 3T magnetic resonance imaging scanner with a 12-channel head coil (Siemens, Erlangen). The diffusion MRI protocol included a B0 field map to correct artifacts and a single shell HARDI SS-EPI sequence along 60 optimized diffusion-weighted directions (*b* = 1,500 *s*/*mm*^2^, 70 slices, matrix = 128 × 128, voxel size = 1.71875 × 1.71875 × 1.7 mm).

The data were pre-processed using BrainVISA/Connectomist 2.0 software (Duclap et al., 2012), with parameters empirically adjusted to achieve a satisfactory reconstruction of all deep white matter bundles that compose the atlas proposed by Guevara et al. (2012). The main sources of artifacts were corrected, and defective slices were discarded. The analytical Q-ball model (Descoteaux et al., 2007) was computed to obtain ODF fields in each voxel. Finally, a whole-brain deterministic streamline tractography was performed, using a T1-based (Guevara et al., 2011a) propagation mask with one seed per voxel at T1 resolution, a maximum curvature angle of 30°, and a forward step of 0.2 mm. On average, the resulting datasets contain around one million fibers per subject. As a post-processing step, all the fibers were resampled using 21 equidistant points (Guevara et al., 2012).

To evaluate the simulator, we used bundles from a Deep White Matter (DWM) bundle atlas (Guevara et al., 2012). This atlas is based on multiple subjects, capturing the variability in shape and position of 36 DWM bundles.

### 2.2 Methods

This section explains the methodology used to develop the fiber bundle simulator, using spline curves and bundle shape parameters. To evaluate the robustness of the simulator, we implemented a validation process (Poo et al., 2023) by simulating bundles from a Deep White Matter (DWM) bundle atlas (Guevara et al., 2012).

To compare the simulated bundles with the reference atlas bundles, two similarity metrics were employed: an inter-bundle distance metric and the percentage of intersection between bundles. In contrast to Poo et al. (2023), where only three fascicles were simulated for the validation process, here we simulated 28 atlas bundles to test the simulator's behavior for a wider variety of bundle shapes.

Finally, we used our algorithm to generate simulated whole-brain tractography datasets, to serve as a ground truth for evaluating the performance of two state-of-the-art fiber clustering algorithms: Quickbundles and FFClust. The performance of both algorithms was evaluated for different distance thresholds, over three datasets with 100, 500, and 1,000 bundles, and different permutations of these simulated data.

#### 2.2.1 Fiber bundle simulator using splines

The simulation of the bundles was performed considering a centroid and the division of the bundle into five cross-sectional regions: the two end regions, the central region, and two intermediate regions. These regions were selected since the radii of the external and central regions of a bundle are important characteristics to take into account when describing the shape of the bundle (Yeh, 2020). In our research, since we only have information from the centroid, we also considered the radii of two intermediate cross-sectional regions. The key parameters for the bundle simulation process were the centroid, which provides an approximate description of the trajectory of the bundle, the radii for each region, and the number of fibers to simulate.

The methodology consists of building a tubular model for the bundle simulation. We used circles at each selected cross-section, centered on their respective centroid points. For creating of a circle, an initial point is selected in its periphery and rotated seven times around its central point, with angles multiples of 45°.

**Figure 1:**
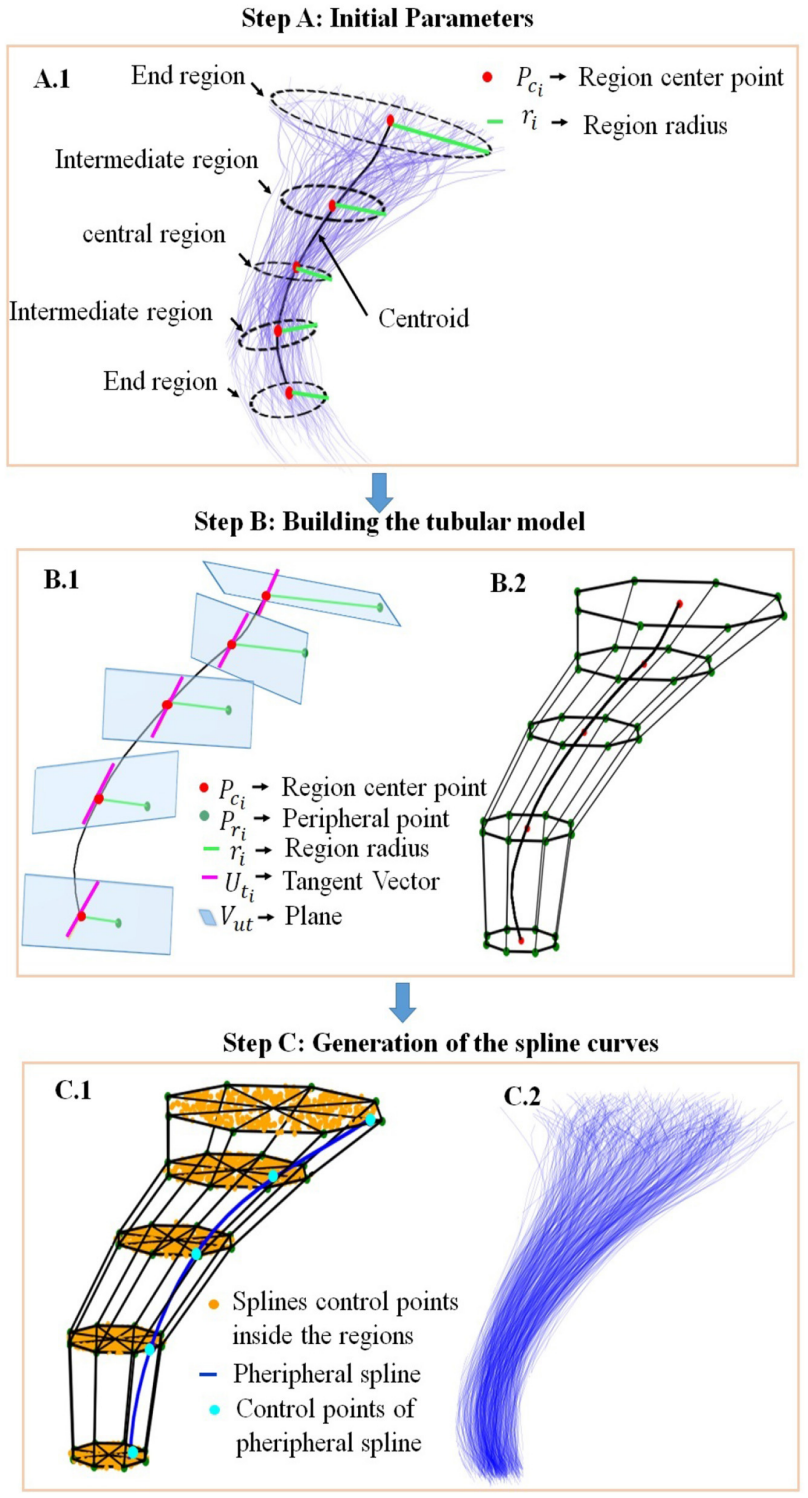
General outline of the bundle simulator based on spline curves, using as example the cortico-spinal tract (CST). Step **(A)** Initial Parameters: **(A.1)** Representation of five cross-sectional regions. Step **(B)** Building the cylindrical model: **(B.1)** Defining the first peripheral point of the circle (*P*_*ri*_), to be rotated around the central point (*P*_*ci*_), **(B.2)** Defining the circle peripheral points, which are the peripheral spline control points. Step **(C)** Generation of the splines curves: **(C.1)** Generation of the spline control points inside the cylindrical model, **(C.2)** Simulation of the bundle, with fibers defined by the splines. The subindex *i* represents the number of the region (from 1 to 5).

The key to obtaining a realistic curve was to use points inside each circle as control points to build the splines. Fourth-order splines were used to ensure the smoothness and continuity of the final curves of the simulated bundle. Figure 1 displays the general outline of the process, and we describe each step next.

##### 2.2.1.1 Step A: initial parameters

We considered five main cross-sectional regions for the bundle simulation: the end regions, the central region, and the intermediate regions, as can be seen in Figure 1A.1. The input parameters for each bundle of the simulation algorithm are the centroid of the bundle, the number of fibers, and the radii of the five cross-sectional regions *r*_*i*_, where the subindex *i* in each parameter represents the number of the region, from 1 to 5. Each radius represents the distance from the farthest fiber of the bundle to the corresponding point on the centroid. To ensure the real dispersion existing at the ends of the bundles, Gaussian noise is optionally added to the first five points of each end, defined by the Gaussian mean and variance as input parameters. To define the centers of the five cross-sectional bundle regions, the algorithm selects five specific points along the centroid (*P*_*ci*_). Since we work with fibers composed of 21 points, we used points with indexes 0, 3, 10, 17, and 20. These points were shown to be representative of the bundle shape in previous work (Labra et al., 2017; Vázquez et al., 2020).

##### 2.2.1.2 Step B: building the tubular model

To construct the tubular model for the bundle shape, five circles are generated around the selected centroid points, to define the five cross-sectional regions. For the creation of a circle, an initial point is selected in its periphery (*P*_*ri*_) and rotated with seven different rotation angles θ, around each selected central point *P*_*ci*_. The rotation angles are multiples of 45°, and are defined in the interval 0–315°, delimiting eight circular sectors of 45°.

Figure 1B.1 illustrates how to obtain the initial peripheral point of each circle (*P*_*ri*_). First, we determine the direction of the bundle centroid at each central point *P*_*ci*_, by calculating the tangent vector ($\vec{U_{ti}}$) to the curve at that point. Then, to define the initial peripheral point of each cross-sectional section *P*_*ri*_, we select a point at a distance *r*_*i*_ from *P*_*ci*_, following the direction of the vector $\vec{P_{ci}P_{ri}}$ (perpendicular to the vector $\vec{U_{ti}}$), and belonging to the plane that contains *P*_*ci*_ defined by $\vec{U_{ti}}$. To ensure that all points are in corresponding positions, they are aligned relative to the first circle point.

For the rotation of *P*_*ri*_ around *P*_*ci*_, a 3 × 3 rotation matrix is constructed (Rodrigues, 1840; Taylor and Kriegman, 1994) based on the corresponding rotation axis $\vec{U_{ti}}=\left(u_{\vec{x}i},u_{\vec{y}i},u_{\vec{z}i}\right)$ and the rotation angle θ, as shown in Equation (1). $\vec{U_{ti}}$ are selected as the axis of rotation for each *P*_*ri*_, which ensures that the rotation faithfully represents the direction of the bundle centroid in each region. Figure 1B.2 shows the tubular model obtained after the initial rotation of the peripheral points of each circle, obtaining a set of eight points defining the circles.


(1) $$\begin{array}{l} R=\left[\begin{array}{ccc} u_{x_{i}}^{2}(1-\cos\theta )+\cos\theta & u_{x_{i}}u_{y_{i}}(1-\cos\theta )-u_{z_{i}}\sin\theta & u_{x_{i}}u_{z_{i}}(1-\cos\theta )+u_{y_{i}}\sin\theta \\ u_{y_{i}}u_{x_{i}}(1-\cos\theta )+u_{z_{i}}\sin\theta & u_{y_{i}}^{2}(1-\cos\theta )+\cos\theta & u_{y_{i}}u_{z_{i}}(1-\cos\theta )-u_{x_{i}}\sin\theta \\ u_{z_{i}}u_{x_{i}}(1-\cos\theta )-u_{y_{i}}\sin\theta & u_{z_{i}}u_{y_{i}}(1-\cos\theta )+u_{x_{i}}\sin\theta & u_{z_{i}}^{2}(1-\cos\theta )+\cos\theta \end{array}\right] \end{array}$$

##### 2.2.1.3 Step C: generation of the spline curves

The previous step generates a tubular model, divided into eight circular sectors along the five cross-sectional sections. For each circular sector, the algorithm generates points following a random uniform distribution, as shown in Figure 1C.1. This arrangement of points generates curves distributed over the entire tubular model. For the representation of brain fibers, fourth-order splines are used since this order is a good approximation to describe the complexity of the fiber shape. Note that for fiber tracking, third-order splines have been used (Wu et al., 2009; Losnegård et al., 2013), but we preferred to use a higher order to guarantee the smoothness of the curves.

To construct the fourth-order splines, it is necessary to have at least five control points. For creating the spline curves, we proceed by sectors. That is, for each spline, we take a point within the same sector for each circle. Figure 1C.1 illustrates the creation of a spline (in blue) and its corresponding control points (in cyan).

To add dispersion of the curve ends, Gaussian noise is optionally added to the points at both ends of the spline. For testing, at each bundle end, we added noise to the first five points of each curve, with a standard deviation, σ, varying in the range of 2.5–3.5 mm. As we use fibers with 21 points, the indexes of these points are 0, 1, 2, 3, 4 for the first end, and 16, 17, 18, 19, 20 for the other end. The addition of noise helps to obtain a more realistic curve with natural variability. The added noise can be modified depending on the desired degree of dispersion. In the example, a set of all the splines obtained using the tubular model is shown in Figure 1C.2.

### 2.2.2 Validating the bundle simulator using a Deep White Matter bundle atlas

The simulator was validated using a DWM bundle atlas (Guevara et al., 2012). As parameters for the simulation, we used the centroid, the radius of each section, and the number of fibers corresponding to each bundle of the original atlas. To determine the centroid of each atlas bundle, first the orientations of the fibers of the bundle were aligned with respect to a reference fiber. The alignment of the bundle fibers is required since when calculating the fibers, these can be stored in memory in any of the two possible orders respecting a reference fiber, direct or inverse order. To align the fibers of a bundle, we first analyze the orientation of each fiber with respect to the reference fiber, by comparing the Euclidean distance between their ending points. If the distance is higher for the opposite ends, the fiber is reoriented by inverting the order of all its points in memory. The reference fiber was identified by selecting fibers with a length >50 mm to remove the short fibers that in general are noisier than longer fibers, and then selecting the fiber with the minimum average distance from all other fibers. Next, the centroid was calculated as the mean of the fibers of the bundle. The radius of each circle was calculated as the mean of the Euclidean distances between the central point of the circle and all the corresponding fiber points of the bundle. The bundle orientation is defined based on the centroid of each atlas bundle. From the 21 points that define the centroid, a spline approximation of the centroid was determined, obtaining the coefficients of the curve equation. With them, to obtain the tangent vectors, we used the equation of the first derivative of the curve and calculated its value at each representative point of the centroid. Then, tangent vectors contain the centroid orientation at these points.

To measure how similar the simulated bundles were to the original atlas bundles, and also to compare the results with a previous simulator (Poo et al., 2023), we used two similarity metrics: the inter-bundle distance and the percentage of intersection between bundles. The inter-bundle distance represents the average distance between the closest fibers of two bundles. The intersection percentage between a pair of bundles is calculated as the percentage of similar fibers between the two bundles. Two fibers are considered similar if the distance between them is < 10 mm (Román et al., 2017; Poo et al., 2023). The calculation of these metrics is based on the use of a distance matrix, where the rows of the matrix represent the distance between the fibers of the simulated bundle and the atlas bundle. As a measure of the distance between the fibers, we used the maximum Euclidean distance between the corresponding points (Román et al., 2017). This measure considers the two possible directions of the fibers, as shown in Equation (2), where *a*_*i*_ and *b*_*i*_ are the indexes of the points in fibers A and B, respectively. *N*_*p*_ corresponds to the number of points of the fiber.


(2) $$\begin{array}{l} d_{ME}\left(A,B\right)=min\left(max_{i}||a_{i}-bi,max_{i}||a_{i}-b_{\left(N_{p}-i\right)}||\right) \end{array}$$

To test the practicability of using a tubular model to represent the bundles, we conducted a short analysis of the shape of the regions of the atlas bundles, by modeling them with elliptical cross-sections (see Supplementary Figures S1, S2). Next, we calculated the ratio between the minor and major radii for the five cross-sectional regions of the analyzed atlas bundles, as well as the average radius ratio. A value closer to 1.0 indicates that both radii are very similar, and consequently the shape will be closer to a circle. As shown in Supplementary Table S1, the radius ratios are, in general, higher than 0.7, with a total average radius ratio of 0.82, which indicates that it is reasonable as a first approach to use a tubular model with circular cross-sections to represent tubular DWM bundles.

For the validation of the simulator, since the algorithm aims to simulate tubular bundles, eight out of the 36 bundles presenting a sheet-like shape were not considered in the analysis. These bundles correspond to the corpus callosum bundles and short cingulum bundles, which present completely different shapes and are usually subdivided into multiple clusters by exploratory clustering algorithms. Hence 28 bundles were simulated, including the left and right versions of the arcuate fasciculus bundles, long cingulum fibers, corticospinal tract, fornix, inferior fronto-occipital fasciculus, inferior longitudinal fasciculus, thalamic radiations, and uncinate fasciculus.

### 2.2.3 Validating fiber clustering algorithms using the bundle simulator

In this study, we generated simulated whole-brain bundle datasets to serve as ground truth for the validation of clustering algorithms. Our methodology involves the following parameters: the bundle centroids, the radii for the circle representing the cross-sectional regions, the number of fibers of each bundle, and the Gaussian noise parameters. The aim is to show the practicality of the proposed simulator in assessing the behavior of two clustering algorithms, QuickBundles (QB) (Garyfallidis et al., 2012) and Fast Fiber Clustering (FFClust) (Vázquez et al., 2020).

The bundle centroids were selected from the whole-brain tractography of a subject from the ARCHI database (Schmitt et al., 2012). This tractography comprises a collection of fibers, each with 21 points in ℝ^3^. First, we filtered out short and noisy fibers by selecting only centroids with a length >50 mm. Next, we chose centroids at a minimum of maximum Euclidean distance of 10 mm and a set of random fibers from the filtered fiber set to act as simulated bundle centroids. We worked with three sets of centroids consisting of 100, 500, and 1,000 centroids.

To select the range of simulation parameters for the circle radii and the number of fibers, we conducted a parameter study based on the bundles of the DWM bundle atlas (Guevara, 2011). As a result, the parameter configuration of the simulation consists of the following ranges for each radius: *r*_1_ and *r*_5_: 8–10 mm (end circles), *r*_2_ and *r*_4_: 6–8 mm (intermediate circles), *r*_3_: 5–7 mm (central circle). For defining the radii, the algorithm works as follows. First, the external radii are selected randomly and separately within their defined range. The intermediate radii are then selected randomly and separately within their corresponding range, but lower than their external radii. Finally, the central radius is randomly selected within its range, but inferior to the radii of the intermediate regions. This procedure aims to avoid irregular shapes when building the tubular model. Then, a Gaussian noise was added, with a σ varying in the range of 2.5–3.5 mm. The number of fibers was chosen from 50 to 300. All random selections are based on a normal distribution.

We generated three simulated ground truth datasets with 100, 500, and 1,000 fiber bundles. We used the *bundles* format, as it allows us to store all bundles in a single binary file (with the extension *.bundlesdata*) and the respective fiber bundle labels in a text file (with the extension *.bundles*). Then we applied QB and FFClust to the simulated tractography datasets using four different distance thresholds.

To apply the algorithms to a simulated dataset, QB only requires the distance threshold (θ_*QB*_) for clustering. It typically utilizes the Minimum Average Direct-Flip (MDF) fiber distance, along with resampling the fibers to 12 points. This algorithm starts by selecting the first fiber in the tractography dataset and placing it in the first cluster. Then it constructs the clusters using the distance threshold (θ_*QB*_) to determine whether a new fiber is assigned to the nearest cluster or initiates a new cluster. We applied QB using four values of θ_*QB*_: 10, 12, 15, and 20 mm.

FFClust is based on four steps. The first step performs a clustering of MiniBatch K-Means over a subset of five points, the two ending points (0, 20), the central point (10), and two intermediate points (3, 17). The values of the number of clusters (*Kp*) for the different points (*Kp*_*end*_, *Kp*_*cent*_, and *Kp*_*inter*_) were obtained for the three datasets using the Elbow method, as proposed by Vázquez et al. (2020). In the second step, the method groups preliminary clusters composed of fibers that share the same point cluster labels computed in the previous step, that is, those sharing the same point clusters for the five points analyzed. In the third step, small clusters are reassigned to the nearest large cluster, considering a distance threshold (*dR*_*max*_). If, at the end of the step, there are still groups consisting of only one or two fibers, these are deemed noisy and discarded. To reduce over-division, the final step merges candidate clusters sharing the central point label, based on a distance threshold (*d*_*Mmax*_). Using a graph, the algorithm identifies and merges clusters within the distance threshold. In the FFClust implementation, the same values are used for both distance thresholds, as using different thresholds in these stages showed no difference in the results of the clusters. For testing, we applied FFClust using the same values as QB for the distance thresholds (*d*_*Rmax*_, *d*_*Mmax*_): 10, 12, 15, and 20 mm.

Another critical issue to consider when evaluating the performance of clustering algorithms is the impact of changing the order of the input elements on the algorithm. To address this consideration, we performed five input data permutations and analyzed the clustering metrics changes. In order to perform these permutations, we used the Fisher–Yates algorithm (Fisher and Yates, 1963), which randomly permutes a sequence of elements. It starts from the last element, randomly chooses an index, and swaps it with the current element. It repeats this process backward through the sequence. This approach guarantees uniformly random permutations and is efficient in terms of time.

Finally, we evaluated the quality of the clusters obtained by the algorithm by comparing them with the ground truth. We use standardized metrics, such as geometric accuracy, precision, recall, and F-measure, to assess the quality of the clusters obtained by clustering algorithms. Furthermore, many research studies have used the Overlap Score (OS) measure proposed by Nepusz et al. (2012). The goal of OS is to assess the degree of agreement between the clusters predicted by any method according to the ground truth clusters (Nepusz et al., 2012; Ji et al., 2014; Hernandez et al., 2017). Equation (3) defines the OS between *C*_*p*_ and *C*_*g*_ corresponding to the cluster predicted by the algorithm and the actual ground truth cluster, respectively.


(3) $$\begin{array}{l} OS\left(C_{p},C_{g}\right)=\frac{|C_{p}\cap C_{g}{|}^{2}}{\left|C_{p}||C_{g}\right|} \end{array}$$

Precision evaluates the proportion of clusters correctly identified and predicted by the algorithm among all predicted clusters. In contrast, Recall measures the fraction of ground truth clusters that are accurately predicted by the algorithm. F-Measure is the harmonic mean of Precision and Recall. Precision, Recall, and F-measure are defined as follows in Equations (4–6).


(4) $$\begin{array}{l} Precision=\frac{TP}{TP+FP}\; \end{array}$$

(5) $$\begin{array}{l} Recall=\frac{TP}{TP+FN}\; \end{array}$$

(6) $$\begin{array}{l} F\text{-}measure=\frac{2*Precision*Recall}{Precision+Recall}\; \end{array}$$

where TP is the number of True Positives, representing the number of clusters matching ground truth clusters with an OS equal to or greater than a threshold. This work uses an OS of 0.8, as suggested by Nepusz et al. (2012). FP represents the number of False Positives and is the difference between the number of clusters predicted by the algorithm and the number of TP. FN or False Negatives refers to the number of ground truth clusters that do not have a match in the clusters predicted by the algorithm. As observed, the TP, FP, and FN values are based on the overlap score.

We also use another two measures: Geometric Accuracy (Acc) and Maximum Matching Ratio (MMR) (Brohée and van Helden, 2006; Nepusz et al., 2012; Hernandez et al., 2017). First, Acc represents the geometric mean of Clustering Wise Sensitivity (Sn) and Positive Predictive Value (PPV). Sn indicates the algorithm's proficiency in identifying fibers within the ground truth, specifically regarding coverage. In contrast, PPV denotes the probability of TP in the cluster predicted by the algorithm. Then Sn, PPV, and Acc can be defined as shown in Equations (7–9).


(7) $$\begin{array}{l} Sn=\frac{\overset{n}{\underset{i=1}{\sum }}\max_{j}t_{ij}}{\overset{n}{\underset{i=1}{\sum }}N_{i}} \end{array}$$

(8) $$\begin{array}{l} PPV=\frac{\overset{m}{\underset{j=1}{\sum }}\max_{i}t_{ij}}{\overset{m}{\underset{j=1}{\sum }}T_{j}} \end{array}$$

(9) $$\begin{array}{l} Acc=\sqrt{S_{n}\times PPV} \end{array}$$

where *t*_*ij*_ is the number of fibers in common between the *i*_*th*_ cluster of the ground truth and the *j*_*th*_ cluster predicted by the algorithm. The number of clusters in the ground truth is *n* and *m* is the number of clusters predicted by the algorithm. *N*_*i*_ is the number of fibers present in the *i*_*th*_ cluster, and $T_{j}=\overset{n}{\underset{i=1}{\sum }}\max_{j}t_{ij}$.

Second, the Maximum Matching Ratio (MMR) (Nepusz et al., 2012; Hernandez et al., 2017) measures the matching proportion of the predicted clusters of the algorithm with the clusters of the ground truth. The MMR penalizes cases where a ground truth cluster is divided into multiple parts in the predicted set. It uses a bipartite graph with weighted edges to measure the quality of the predicted set relative to the ground truth set. The MMR score reflects the sum of edge weights divided by the number of clusters in the ground truth. Edges are weighted on the basis of the overlap score between the clusters. The MMR is computed by summing all OS values of TP-predicted clusters divided by the number of ground truth clusters.

## 3 Results

All experiments were performed on a machine equipped with a 3.0 GHz Intel(R) Core(TM) i7-9700 CPU and 32 GB of RAM, using Windows 10 Pro (64-bits). We describe the results for the bundle simulator using 28 DWM bundles, followed by the experiments for the validation of fiber clustering algorithms using the bundle simulator.

### 3.1 Validating the bundle simulator using the DWM bundle atlas

In order to validate the proposed simulator, we generated 28 bundles of the DWM bundle atlas with different shapes and number of fibers.

We used two metrics to compare the simulator results with the atlas bundles: the inter-bundle distance and the percentage of intersection between bundles. These metrics, based on the maximum Euclidean distance between fibers (Equation 2), allow us to evaluate the accuracy of the representation of the bundle shape. Table 1 presents detailed results for both metrics.

**Table 1:** Metrics results between the simulated bundles and the bundles of the atlas for the proposed simulator and the previous simulator, based on exponential curves (Poo et al., 2023): previous simulator I (simulation using five centroids), previous simulator II (simulation using one centroid), inter-bundle distance ± standard deviation (mm) (ID ± STD) and intersection percentage between bundles (%) (IP).

	**Previous simulator (I)**		**Previous simulator (II)**		**Proposed simulator**	
**Atlas bundles**	**ID** ± **STD (mm)**	**IP (%)**	**ID** ± **STD (mm)**	**IP (%)**	**ID** ± **STD (mm)**	**IP (%)**
Anterior left arcuate	10.5 ± 4.4	61.2	10.6 ± 4.2	59.1	8.2 ± 1.1	96.4
Anterior right arcuate	10.6 ± 3.9	60.7	10.9 ± 4.2	58.6	8.2 ± 1.0	97.9
Left arcuate	15.0 ± 4.2	21.4	17.3 ± 5.4	7.9	10.5 ± 2.2	44.8
Right arcuate	13.9 ± 4.8	25.2	15.9 ± 4.7	8.5	10.8 ± 2.2	39.9
Posterior left arcuate	8.3 ± 2.9	81.3	8.7 ± 3.7	68.9	6.6 ± 1.0	100.0
Posterior right arcuate	8.3 ± 2.9	77.0	8.9 ± 3.5	67.2	6.7 ± 0.9	100.0
Left cingullum temporal fibers	7.5 ± 2.5	81.5	9.8 ± 4.6	57.3	7.9 ± 1.6	86.4
Right cingullum temporal fibers	7.9 ± 2.7	81.5	10.1 ± 4.9	57.3	7.2 ± 1.4	89.0
Left corticospinal tract	8.2 ± 2.1	82.0	13 ± 7.2	47.4	8.1 ± 1.6	87.3
Right corticospinal tract	6.8 ± 3.1	85.6	12.8 ± 7.4	52.4	8.0 ± 1.6	87.5
Left fornix	9.1 ± 3.6	68.5	13.6 ± 5.4	31.4	10.6 ± 1.8	32.9
Right fornix	9.2 ± 3.8	68.6	13.8 ± 5.8	32.9	10.8 ± 1.7	30.0
Left inferior fronto-occcipital	14.2 ± 6.4	31.9	16.1 ± 7.7	8.9	11.9 ± 1.7	13.2
Right inferior fronto-occcipital	14.1 ± 6.1	27.11	16.5 ± 7.9	8.2	11.5 ± 1.5	15.5
Left inferior longitudinal	11.0 ± 4.8	45.4	16.6 ± 7.8	20.8	10.3 ± 1.8	48.2
Right inferior longitudinal	11.9 ± 5.4	46.4	17.0 ± 5.4	20.8	10.2 ± 1.8	49.6
Left frontal thalamic radiations	9.4 ± 3.8	62.3	10.5 ± 4.2	55.9	7.9 ± 1.7	88.5
Right frontal thalamic radiations	10.1 ± 5.2	56.5	10.9 ± 5.8	50.1	8.1 ± 1.8	85.4
Left superior motor thalamic radiations	7.1 ± 2.6	86.2	10.4 ± 6.3	59.7	7.1 ± 1.1	99.3
Right superior motor thalamic radiations	7.2 ± 3.1	84.8	9.4 ± 5	66.4	7.2 ± 1.3	98.2
Left occipital thalamic radiations	9.5 ± 4.5	71.1	11.5 ± 7.1	55.4	7.6 ± 1.6	90.4
Right occipital thalamic radiations	10.4 ± 5.0	64.0	11.7 ± 6.8	55.4	7.7 ± 1.5	92.8
Left superior parietal thalamic radiations	7.3 ± 2.9	81.9	8.9 ± 5.1	73.2	6.3 ± 1.1	100.0
Right superior parietal thalamic radiations	7.5 ± 3.2	79.8	9.4 ± 5.8	66.4	7.5 ± 1.2	100.0
Left temporal thalamic	8.5 ± 3.3	75.4	10.9 ± 5.5	50.8	7.2 ± 1.6	93.8
Right temporal thalamic	8.5 ± 2.7	75.4	10.9 ± 4.8	50.8	7.2 ± 1.4	95.4
Left uncinate	9.6 ± 2.6	65.2	11.1 ± 5.2	54.8	8.4 ± 1.2	90.5
Right uncinate	9.3 ± 3.9	67.3	10.3 ± 4.1	56.3	8.6 ± 1.2	88.6
**Mean values**	**9.7** **±** **3.8**	**64.8**	**11.9** **±** **5.7**	**46.5**	**8.5** **±** **1.5**	**76.5**

The mean values of ID ± STD (mm) and IP (%) for the three simulators are highlighted in bold.

**Figure 2:**
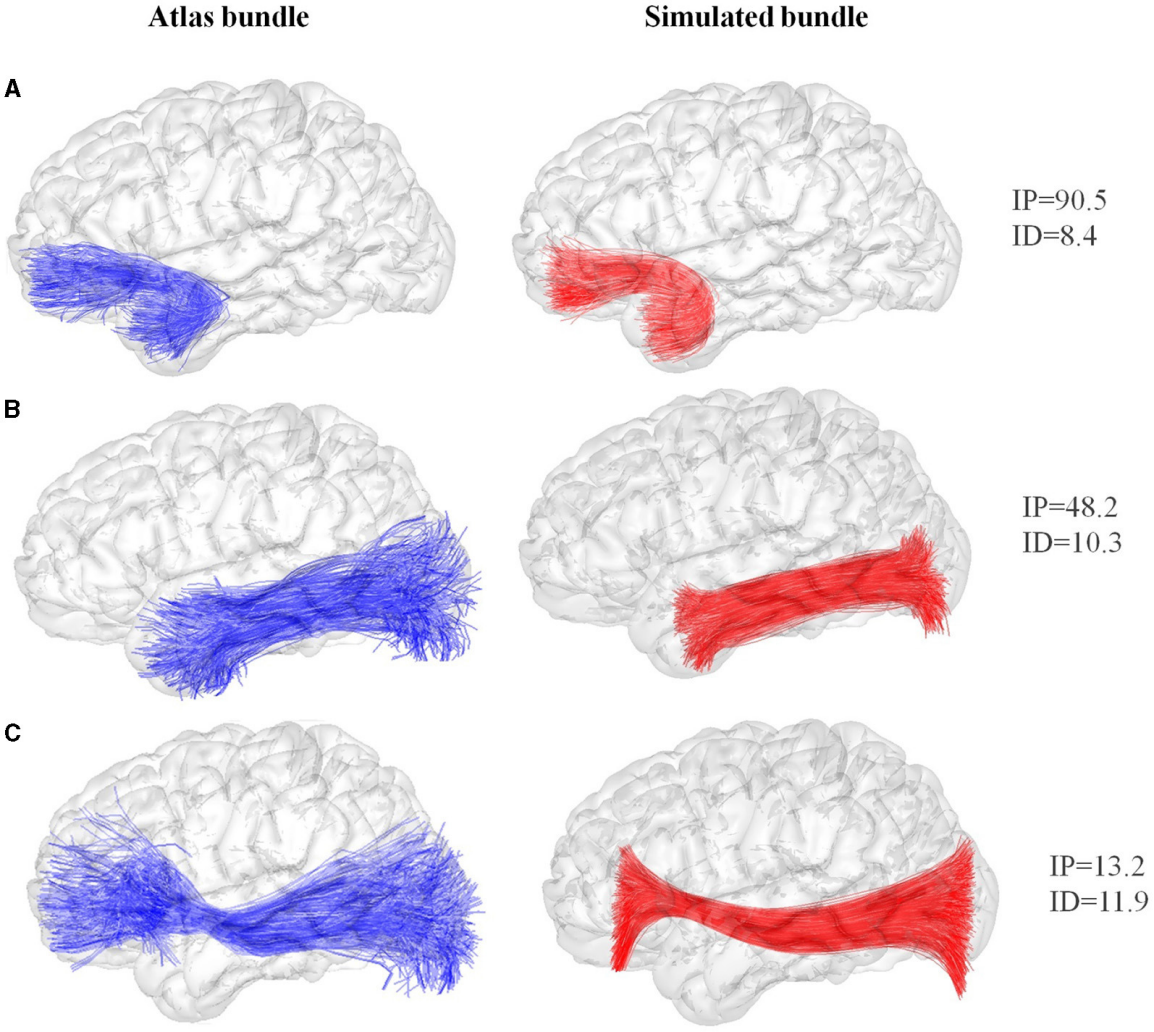
Examples of bundle simulation based on the DWM bundle atlas (Guevara et al., 2012). Original bundles of the atlas are displayed at the left (blue), and their corresponding simulated bundles are shown at the right (red). **(A)** The left uncinate fasciculus, **(B)** the left inferior longitudinal and **(C)** the inferior fronto-occipital. IP, intersection percentage between bundles (%); ID, inter-bundle distance (mm).

Figure 2 shows three simulation results corresponding to bundles of the atlas with different shapes: the left uncinate fasciculus, the left inferior longitudinal fasciculus, and the inferior fronto-occipital fasciculus. The first simulated bundle, shown in Figure 2A, achieved a good representation quality with a bundle intersection percentage of 90.5% and an inter-bundle distance of 8.4 ± 1.2 mm. Figure 2B shows a medium-quality result, with an intersection percentage of 48.2% and an inter-bundle distance of 10.3 ± 1.8 mm. Figure 2C shows an example of low-quality result, with an intersection percentage of 13.2% and an inter-bundle distance of 11.9 ± 1.7 mm. The result of medium or low quality is due to bundles whose ends are very dispersed having variable radii. Therefore, a representation using a single centroid and a mean radius of the fibers to the centroid may only partially capture the bundle's shape. Supplementary Figures S3, S4 show the remaining simulated bundles of the atlas.

We also performed simulations using our previous simulator based on exponential curves. As in Poo et al. (2023), we simulated the atlas bundles by decomposing them into five sub-bundles. However, this strategy utilizes five centroids; hence, to have a more fair comparison of bundle models, we also simulated bundles with exponential curves using only one centroid. Table 1 presents the comparison results.

The results show a good performance of the proposed simulator, with a mean inter-bundle distance (ID) (and standard deviation) of 8.5 ± 1.5 mm and a mean intersection percentage (IP) of 76.5%. The method outperforms the previous simulator based on five centroids, with average metrics of 9.7 ± 3.8 mm and 64.8%, respectively, and based on only one centroid, with average metrics of 11.9 ± 5.7 mm and 46.5%, respectively. These results show that the simulator can successfully reproduce most bundles. Specifically, 20 of the 28 simulated bundles had a percentage of intersection >85% with the corresponding atlas bundle. Nonetheless, some bundles, such as the left and right inferior fronto-occipital fasciculus, were poorly simulated. These bundles have complex shapes with spread ends, which makes it difficult to simulate them using the information given by only one centroid. Table 1 shows that the previous simulator using five sub-bundles obtained better intersection percentages for these specific fascicles, but when using only one centroid, it presented a lower performance. It is important to note that the calculation of the bundle centroid also affects the simulation results. Here, we used the most common centroid formula, based on the mean of the bundle corresponding points, which naturally creates centroids that do not extend over the entire length of the bundles.

Additionally, Supplementary Table S1 shows the simulation results obtained using the preliminary model based on elliptical cross-sections, compared to the proposed tubular model. Supplementary Figure S5 the three fascicles displayed in Figure 2 for the two models. In Supplementary Table S1 we can observe that improvements in IP and ID were found only in eight bundles (left and right cortical spinal tracts, left and right fornices, left and right occipital thalamic radiations, and left and right uncinate fasciculus), with comparable or inferior results for the remaining bundles. Hence, future work could improve this model, by finding better strategies to define the radii of the ellipses.

### 3.2 Validating fiber clustering algorithms using the bundle simulator

Using our tubular model, we simulated three whole-brain datasets with 100, 500, and 1,000 bundles, which served as ground truth to evaluate the performance of two fiber clustering algorithms. Table 2 shows the main characteristics of the ground truth for the different number of bundles, including the number of centroids used for the simulation and the minimum and mean distance between them. Supplementary Figure S6 shows the centroid sets. Table 2 also lists the minimum and maximum number of fibers per bundle, the total number of fibers of the ground truth, and the number of crossing bundles. A more detailed view of the simulated bundle datasets is shown in the Supplementary Figures S7–S9.

**Table 2:** Ground truth characterization: the number of centroids used for the simulation, the minimum and mean distance between centroids, the minimum and maximum number of fibers in the bundles, the total number of fibers in the ground truth, and the number of fascicles with crossing.

**Ground truth characterization**			
Number of centroids	100	500	1,000
Minimum distance between centroids (mm)	13.23	10.16	10.01
Mean distance between centroids (mm)	90.03	91.69	90.81
Minimum number of fibers per bundle	50	50	50
Maximum number of fibers per bundle	300	300	300
Total number of fibers	19,052	87,728	176,300
Number of crossed bundles	15	274	619

**Figure 3:**
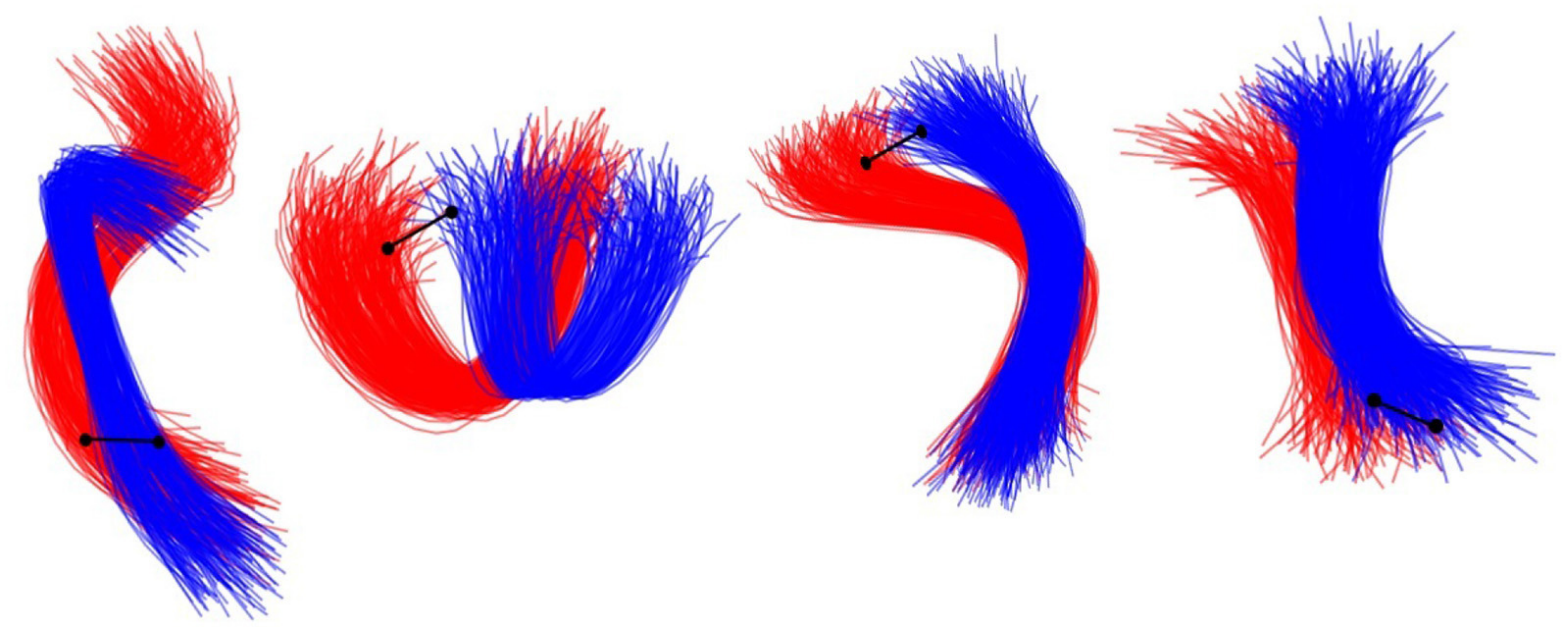
Examples of different types of crossings existing between two bundles in the ground truth datasets. The red and the blue bundle represents two different bundles in the ground truth. The black line represents a 10 mm distance marker.

Given that simulated bundles contain the generated fibers around their centroids, some bundles can contain fibers that become very close to the fibers of other bundles. We denote these bundles as crossing bundles. Thus, we define crossing bundles as those bundles that contain fibers that are at a distance below 10 mm from the fibers of other bundles. Figure 3 displays different types of crossing bundles in the ground truth.

We applied the QB and FFClust algorithms with different distance thresholds of 10, 12, 15, and 20 mm to these simulated datasets. As mentioned in the previous section, for the FFClust algorithm, we also use the Elbow method to set the number of clusters per fiber point. Supplementary Figure S10 shows the Elbow curves corresponding to the three ground truth datasets.

**Figure 4:**
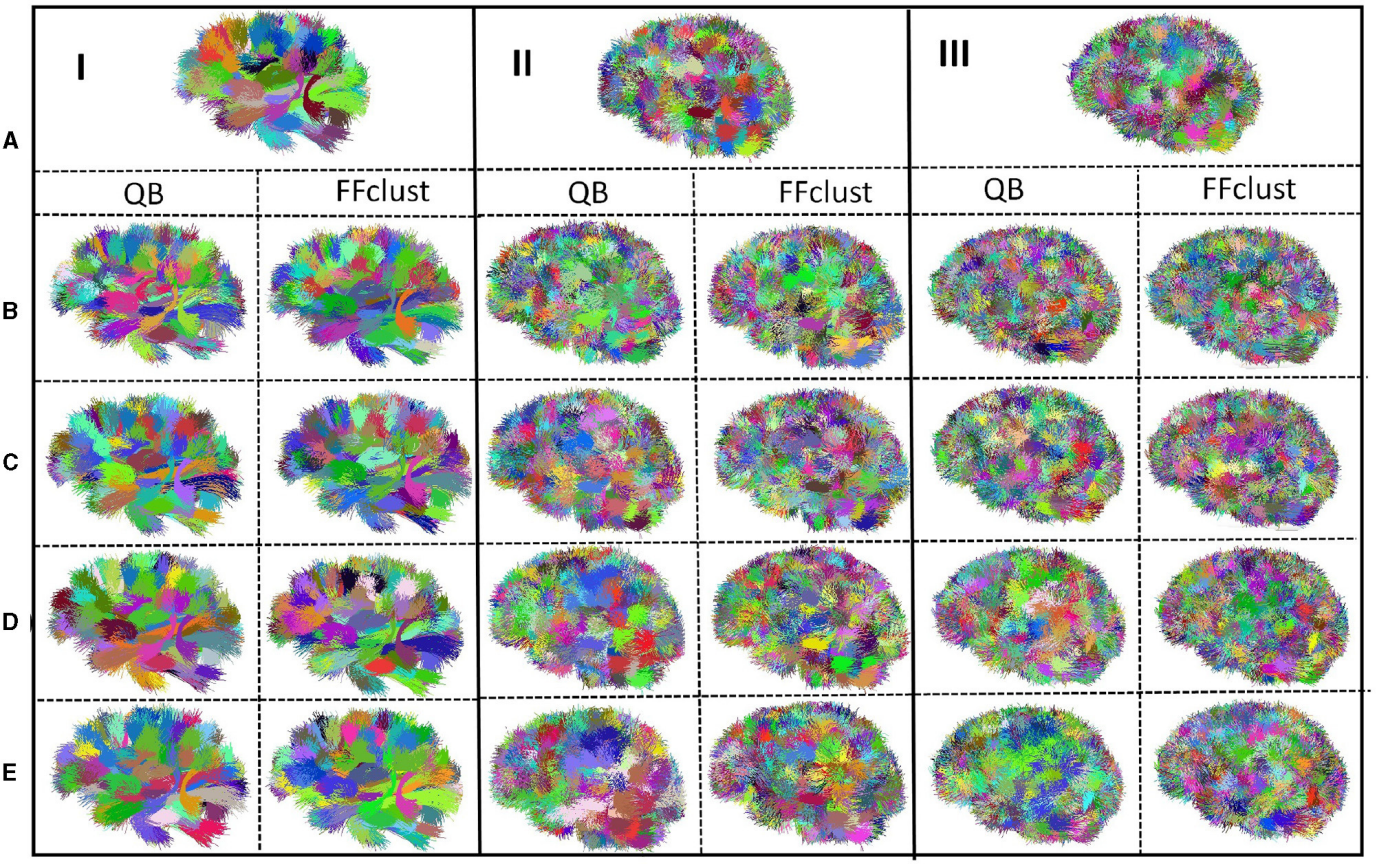
QB and FFClust fiber clustering results obtained for simulated tractography datasets with 100 bundles (I), 500 bundles (II) and 1,000 bundles (III): **(A)** Original simulated bundles. Clusters obtained by QB and FFClust for different thresholds: **(B)** 10 mm, **(C)** 12 mm, **(D)** 15 mm, **(E)** 20 mm.

Figure 4 shows the clusters obtained by both algorithms for the different distance thresholds. As observed, there is a higher crossing between the bundles of datasets II and III (500 and 1,000 fascicles), corresponding to the information shown in Table 2, due to the higher number of bundles used. Also, the behavior of both algorithms is quite similar, increasing the size of the clusters as the distance threshold increases.

To evaluate the quality of the clusters obtained by the algorithms, we calculated the five measures described earlier: Accuracy (Acc), Precision, Recall, F-measure, and Maximum Matching Ratio (MMR). The results of the different measures for both algorithms can be seen in Tables 3, 4 for the simulated tractography datasets with 100, 500, and 1,000 bundles, respectively.

**Table 3:** Metrics values to evaluate the performance of QB algorithm for the three simulated tractography datasets (100, 500, and 1,000 bundles).

**Original clusters**	**100**				**500**				**1,000**			
**Thresholds (mm)**	**10**	**12**	**15**	**20**	**10**	**12**	**15**	**20**	**10**	**12**	**15**	**20**
Algorithm clusters	168	111	91	76	748	468	328	199	1432	890	572	283
Accuracy	0.87	0.95	0.93	0.87	0.81	0.86	0.81	0.66	0.79	0.81	0.73	0.56
Precision	0.31	0.72	0.85	0.75	0.21	0.52	0.55	0.36	0.21	0.48	0.48	0.30
Recall	0.51	0.79	0.77	0.57	0.31	0.48	0.36	0.14	0.29	0.42	0.28	0.08
F-Measure	0.38	0.75	0.81	0.65	0.25	0.50	0.43	0.20	0.24	0.45	0.35	0.13
Maximum Matching Ratio	0.49	0.78	0.77	0.56	0.30	0.47	0.35	0.14	0.28	0.40	0.27	0.08

In blue, the metrics for the threshold with the best performance are highlighted.

**Table 4:** Metrics values to evaluate the performance of FFClust algorithm for the three simulated tractography datasets (100, 500, and 1,000 bundles).

**Original clusters**	**100**				**500**				**1,000**			
**Thresholds**	**10**	**12**	**15**	**20**	**10**	**12**	**15**	**20**	**10**	**12**	**15**	**20**
Algorithm clusters	176	155	126	117	1193	978	797	688	2722	2171	1,719	1527
Accuracy	0.93	0.94	0.95	0.93	0.79	0.82	0.82	0.81	0.73	0.76	0.78	0.76
Precision	0.43	0.51	0.66	0.67	0.11	0.18	0.26	0.25	0.06	0.12	0.18	0.18
Recall	0.76	0.79	0.83	0.78	0.27	0.35	0.41	0.34	0.16	0.25	0.32	0.27
F-Measure	0.55	0.62	0.73	0.72	0.16	0.24	0.32	0.29	0.09	0.16	0.23	0.21
Maximum Matching Ratio	0.73	0.76	0.81	0.76	0.24	0.33	0.39	0.33	0.15	0.23	0.30	0.26

In blue, the metrics for the threshold with the best performance are highlighted.

Tables 3, 4 list the number of clusters predicted by the algorithms. Supplementary Tables S2, S3 show the values of TP, FP, FN, PPV, and Sn. In general, both algorithms performed well as distance thresholds increased. While QB tends to split less, FFClust tends to merge less, behaviors that are expected for both algorithms, and consistent for the different number of clusters. As QB has as a unique input parameter, the distance threshold (θ_*QB*_), its behavior only depends on this value. As the threshold increases, fewer clusters are created and fibers located more distant to the cluster centroids are added to the clusters. If θ_*QB*_ is too big, groups of fibers with different shapes will be clustered together forming bigger and thicker clusters. On the other hand, FFClust has more parameters that have an impact on the final results. The values of the number of clusters (*Kp*) for the different points in the first step (*Kp*_*end*_, *Kp*_*inter*_, and *Kp*_*cent*_) are the most important, since they impact the size of the point clusters. A higher number of point clusters will produce smaller preliminary fiber clusters. The two distance thresholds also impact the clustering results, by reassigning small clusters to big clusters (*d*_*Rmax*_) and merging clusters sharing the central point cluster (*d*_*Mmax*_). Therefore, higher distance thresholds will also produce larger and thicker clusters, but to a lesser extent than QB, so that it is less likely to merge fibers with different shapes, but conversely, more likely to over-subdivide clusters.

As seen in Tables 3, 4 and Figure 4, QB had its best performance for a threshold of 12 mm for all the ground truth datasets, while FFClust performed best for a threshold of 15 mm. As the number of bundles increases, quality measures decrease, which is expected due to the increase in bundle crossings. These crossings might confuse the clustering algorithms.

The maximum value of the measures was obtained for the ground truth of 100 fascicles for both algorithms, reaching an Accuracy of 0.95 in both cases. Furthermore, QB achieves higher Accuracy than FFClust in the larger datasets. The Accuracy values decrease with larger datasets for both algorithms, and QB is more sensitive to the distance threshold than FFClust in all datasets. These values are achieved because both algorithms present a good proportion of true positives (TP), as seen in Supplementary Tables S2, S3. Similarly, the precision reaches close values for both algorithms, with 0.72 for QB and 0.66 for FFClust for the smaller dataset. However, the precision values are more affected in FFClust with the increasing number of bundles, mainly due to an increase in the false positives (see Supplementary Table S3). In contrast, FFClust presents a higher Recall than QB in the smallest dataset and lower than in the largest datasets.

In the last experiment, we assessed the algorithm's ability to detect crossing bundles accurately. We computed the total count of TP clusters (those with *OS*≥0.8) of each algorithm output corresponding to a crossing bundle in the ground truth for each ground truth dataset and threshold. Then, we computed the percentage of crossing bundle recovery per algorithm by dividing such total count by the total number of crossings in the bundle of the ground truth. The results presented in Table 5 illustrate that the algorithms' optimal performance in terms of crossing recovery aligns with the thresholds identified as the best performers in Tables 3, 4 (12 mm for QB and 15 mm for FFClust). Additionally, across most ground truth datasets and thresholds, FFClust outperforms QB, consistent with expectations stemming from differing grouping methodologies.

**Table 5:** Performance of the QB and FFClust algorithms in retrieving crossing bundles for different thresholds (10, 12, 15, and 20 mm) and three ground truth datasets (100, 500, and 1,000 bundles): percentage of clusters recovered by the algorithm (% crossing clusters recov).

**Original clusters**	**100**				**500**				**1,000**			
**Crossing clusters**	**15**				**274**				**619**			
**Thresholds**	**10**	**12**	**15**	**20**	**10**	**12**	**15**	**20**	**10**	**12**	**15**	**20**
% crossing clusters recov QB	33.33	40.00	20.00	0.00	19.71	19.71	6.20	1.46	19.55	19.55	5.82	0.65
% crossing clusters recov FFClust	66.67	66.67	66.67	40.00	19.71	20.80	23.36	12.41	9.21	12.76	14.38	8.89

The values of the F-measure, which represent a trade-off between precision and recall metrics, are affected by the increase in the number of fascicles. In the case of MMR, which provides a measure of the degree of similarity between the clusters predicted by the algorithm and the ground truth, it also shows similar behavior in both algorithms, with FFClust exceeding the MMR values only for the smaller dataset.

**Figure 5:**
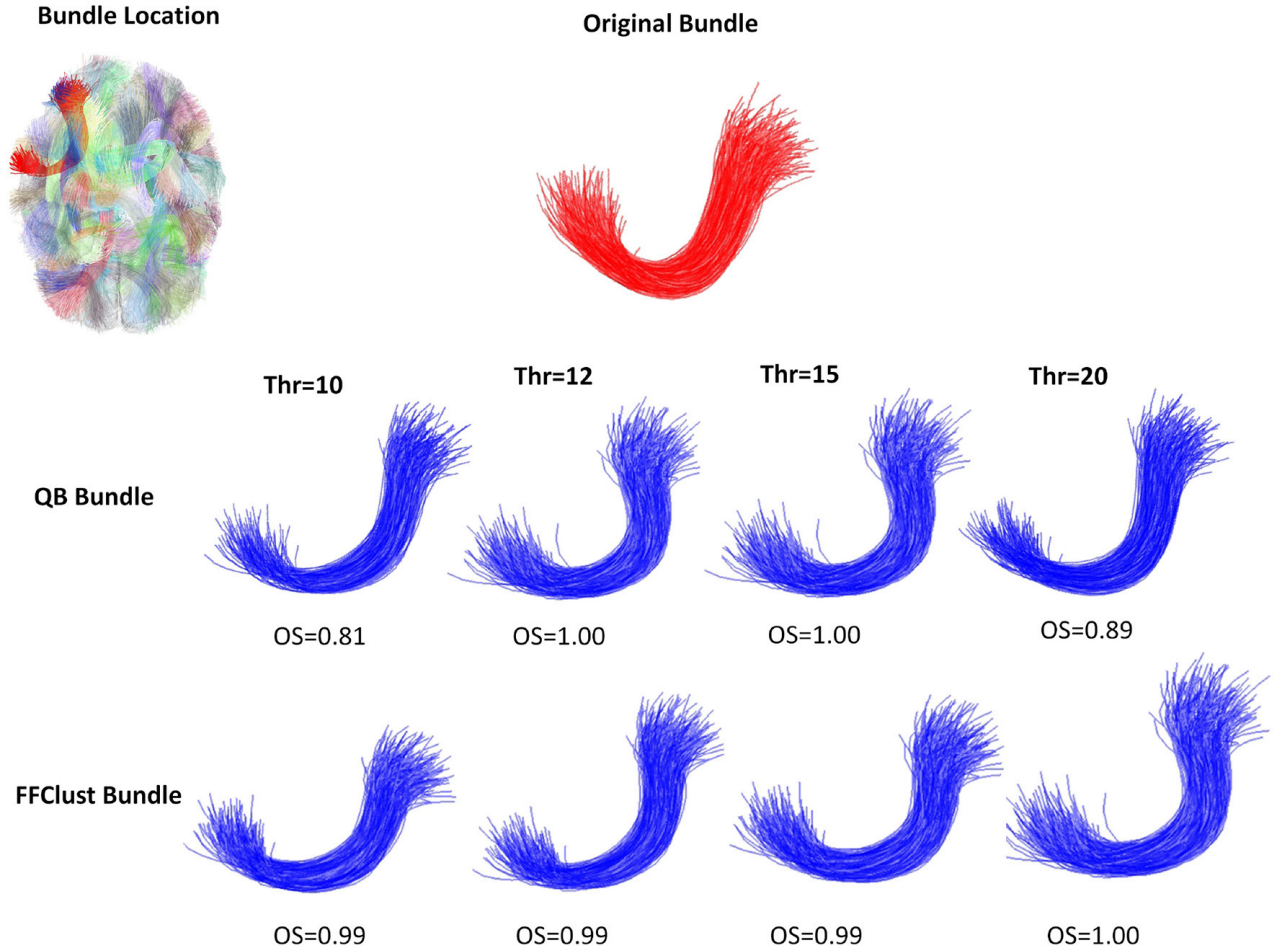
An example of a good performance of QB and FFClust clustering algorithms when retrieving a cluster from the ground truth. The upper part of the figure shows the original bundle and its location in the brain. In the middle and bottom parts, it can be observed the bundle predicted by QB and FFClust for the different thresholds and their corresponding OS values.

**Figure 6:**
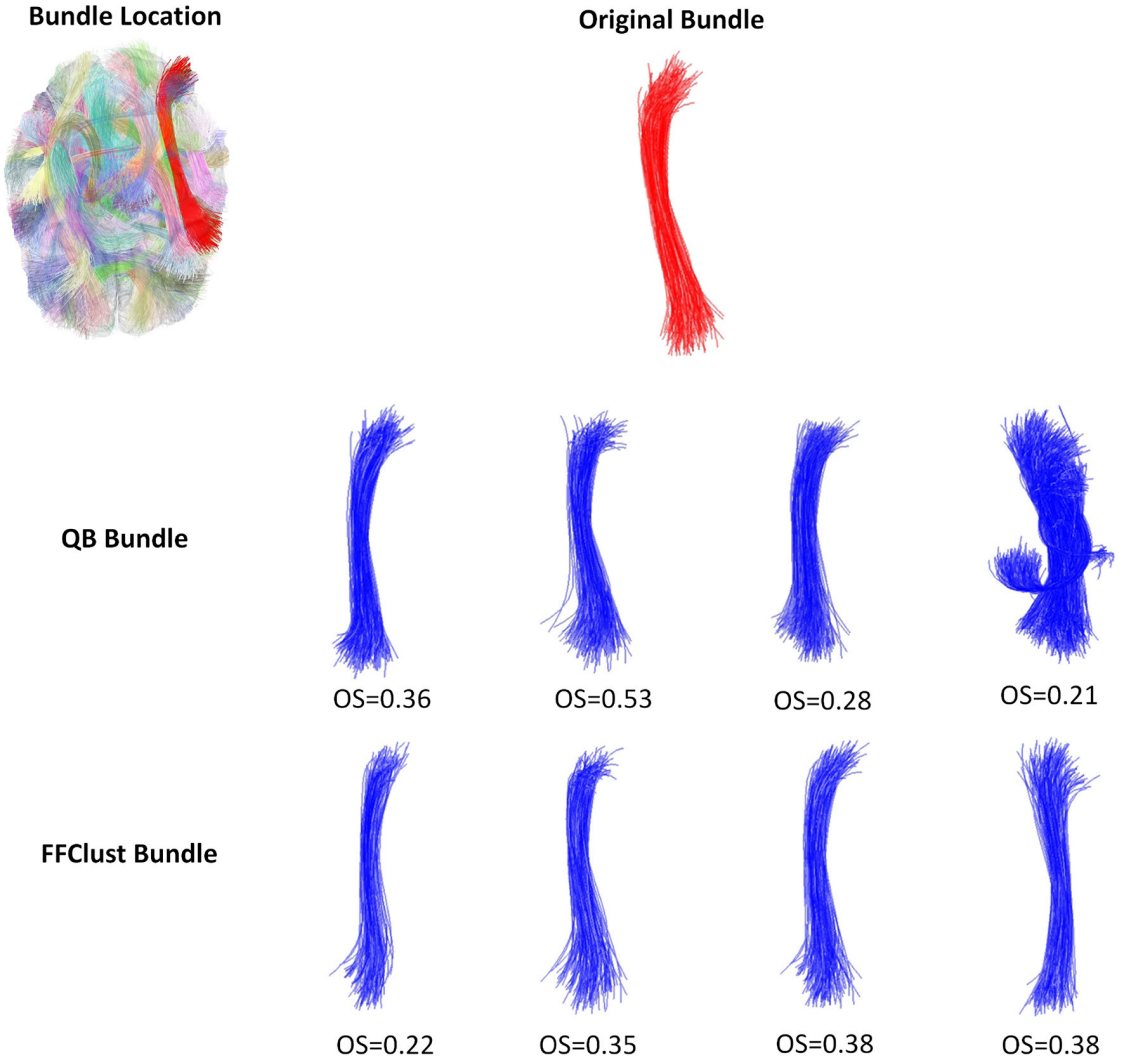
An example of a bad performance of the QB and FFClust clustering algorithms when retrieving a cluster from the ground truth. The upper part of the figure shows the original bundle and its location in the brain. In the middle and bottom parts, it can be observed the bundle predicted by QB and FFClust for the different thresholds and their corresponding OS values.

**Figure 7:**
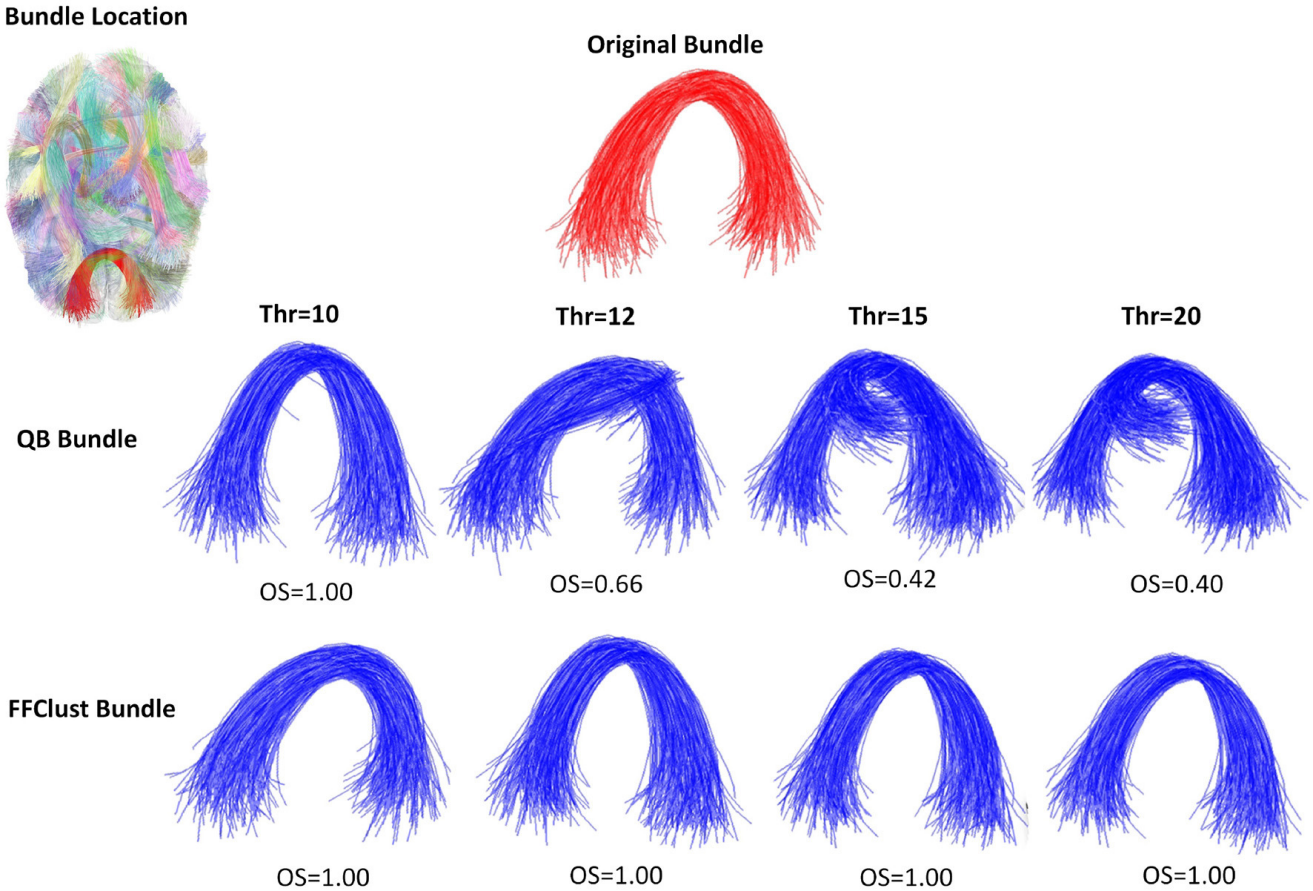
An example of the performance of the QB and FFClust clustering algorithms when retrieving a cluster from the ground truth, where FFClust has better performance than QB. The upper part of the figure shows the original bundle and its location in the brain. In the middle and bottom parts, it can be observed the bundle predicted by QB and FFClust for the different thresholds and their corresponding OS values.

**Figure 8:**
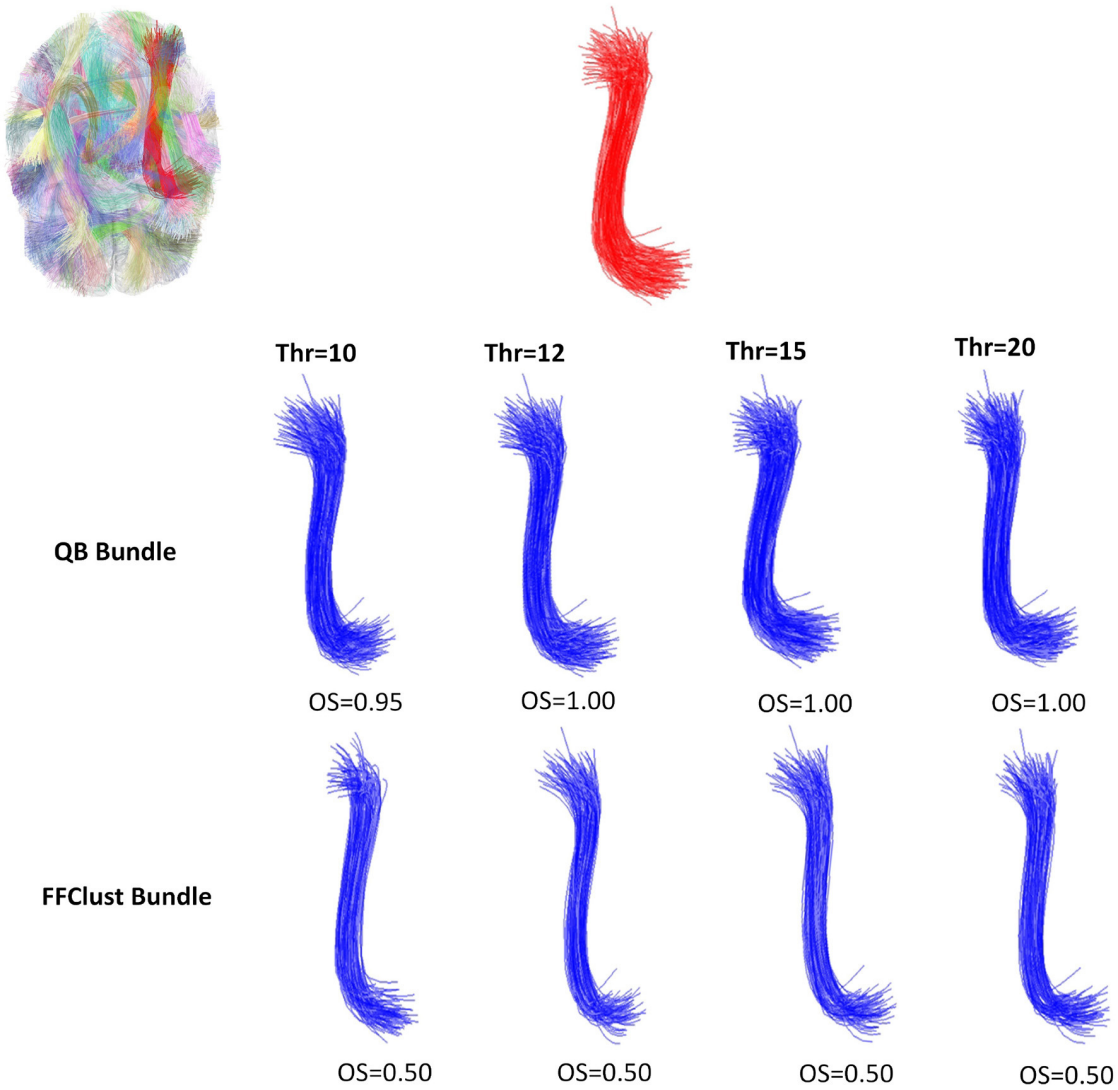
An example of the performance of the QB and FFClust clustering algorithms when retrieving a cluster from the ground truth, where QB has better performance than FFClust. The upper part of the figure shows the original bundle and its location in the brain. In the middle and bottom part, it can be observed the bundle predicted by QB and FFClust for the different thresholds and its corresponding OS values.

Figures 5–8 illustrate examples of the performance of the algorithms in retrieving specific ground truth clusters. At the top of each figure, the original bundle of the ground truth dataset is presented along with its location in the brain. The middle section shows the bundle retrieved by QB for different thresholds, while in the bottom part the same is presented but for the FFClust algorithm. In each figure are included the OS values obtained by the algorithm bundle compared to the ground truth considering different thresholds.

Figure 5 displays a successfully recovered bundle by both algorithms, with OS values >0.8. In contrast, Figure 6 shows a poor performance of the algorithms in retrieving the bundle. QB tends to merge the bundle with others as the threshold increases, while FFClust splits the bundle into several bundles, recovering only a small percentage of fibers of the original bundle. Figure 7 shows an example where FFClust obtains higher OS values than QB. This is because FFClust can preserve the fascicle for different distance thresholds, unlike QB, which tends to merge clusters as the threshold increases. The explanation is that FFClust does not only depends on the distance thresholds, but also on the number of point clusters in its first step. Figure 8 presents the prediction of a ground truth fascicle where QB outperforms FFClust in recovery, with a similar behavior for all thresholds. This is due to the tendency of FFClust to split fascicles, especially when they are large. Finally, Supplementary Figure S11 and Supplementary Table S4 show the results for a newer version of the QB algorithm called QBX (Garyfallidis et al., 2016). This algorithm is 95% faster than QB and the values of the metrics for the highest threshold are the same for both algorithms. However, for the remaining distance thresholds there is a degradation in the quality of the results with respect to those obtained by QB, due to their tendency to subdivide the fascicles along the smaller thresholds and to create clusters of fewer fibers.

Another important condition to consider when evaluating the performance of clustering algorithms is to determine the sensitivity of the algorithms regarding the order of the input data elements. To evaluate this, we performed five random permutations of the input data for each dataset. We computed the metrics averages for the algorithms against the permutations. Table 6 presents the results for QB and Table 7 for FFClust. Supplementary Tables S5, S6 report the variations of the TP, FP, FN, PPV, and Sn measures, used to calculate the other metrics. In general, the measure values are similar to those obtained in the original data without permutation, with low standard deviations. Note that the mean measures for all the permuted datasets presented the best performance for the same thresholds as for the original input data, that is, 12 mm threshold for QB and 15 mm for FFClust. This shows that the algorithms are stable at different permutations of the input data.

**Table 6:** Performance of QB algorithm for five random permutations of the three original simulated tractography datasets.

**Original clusters**	**100**				**500**				**1,000**			
**Thresholds**	**10**	**12**	**15**	**20**	**10**	**12**	**15**	**20**	**10**	**12**	**15**	**20**
Algorithm clusters	168.33 ± 4.04	115.33±3.79	89.67 ± 0.58	70.67 ± 2.08	747.67 ± 10.21	466.67±3.06	311.00±4.36	182.00 ± 4.36	1436.00 ± 13.08	868.33±8.50	528.67 ± 4.51	262.33 ± 3.06
Accuracy	0.87 ± 0.02	0.94±0.01	0.93 ± 0.01	0.84 ± 0.02	0.79 ± 0.01	0.84±0.00	0.79±0.01	0.64 ± 0.01	0.77 ± 0.00	0.79±0.00	0.70 ± 0.00	0.54 ± 0.00
Precision	0.28 ± 0.04	0.67±0.00	0.83 ± 0.04	0.67 ± 0.04	0.21 ± 0.01	0.48±0.00	0.52±0.02	0.30 ± 0.02	0.21 ± 0.01	0.44±0.02	0.43 ± 0.01	0.24 ± 0.01
Recall	0.46 ± 0.06	0.76±0.03	0.75 ± 0.04	0.48 ± 0.04	0.31 ± 0.02	0.45±0.01	0.32±0.01	0.11 ± 0.01	0.30 ± 0.01	0.38±0.01	0.23 ± 0.01	0.06 ± 0.00
F-Measure	0.35 ± 0.05	0.71±0.01	0.79 ± 0.04	0.56 ± 0.04	0.25 ± 0.01	0.46±0.00	0.40±0.01	0.16 ± 0.01	0.25 ± 0.01	0.41±0.02	0.30 ± 0.01	0.10 ± 0.01
Maximum Matching Ratio	0.45 ± 0.06	0.75±0.02	0.74 ± 0.04	0.47 ± 0.04	0.30 ± 0.01	0.43±0.00	0.32±0.01	0.11 ± 0.01	0.28 ± 0.01	0.37±0.01	0.22 ± 0.01	0.06 ± 0.00

The table shows the mean and standard deviation values of all metrics. In blue, the metrics for the threshold with the best performance are highlighted.

**Table 7:** Performance of FFClust algorithm for five random permutations of the original simulated tractography dataset.

**Original clusters**	**100**				**500**				**1,000**			
**Thresholds**	**10**	**12**	**15**	**20**	**10**	**12**	**15**	**20**	**10**	**12**	**15**	**20**
Algorithm clusters	189.00 ± 8.89	155.67 ± 5.03	131.00±4.58	121.33 ± 1.53	1186.33 ± 9.07	964.33 ± 4.73	773.33±16.8	672.67 ± 8.33	2650.33 ± 6.11	2150.00 ± 18.36	1723.67±13.43	1495.00 ± 7.94
Accuracy	0.93 ± 0.01	0.94 ± 0.01	0.95±0.01	0.94 ± 0.01	0.79 ± 0.00	0.82 ± 0.00	0.83±0.00	0.81 ± 0.00	0.73 ± 0.00	0.76 ± 0.00	0.78±0.00	0.76 ± 0.00
Precision	0.36 ± 0.03	0.49 ± 0.02	0.60±0.04	0.62 ± 0.03	0.12 ± 0.01	0.18 ± 0.00	0.25±0.02	0.26 ± 0.01	0.06 ± 0.00	0.11 ± 0.01	0.18±0.01	0.18 ± 0.01
Recall	0.67 ± 0.02	0.76 ± 0.02	0.79±0.04	0.75 ± 0.03	0.27 ± 0.01	0.36 ± 0.01	0.39±0.02	0.35 ± 0.01	0.16 ± 0.00	0.25 ± 0.01	0.31±0.01	0.27 ± 0.01
F-Measure	0.47 ± 0.03	0.59 ± 0.02	0.68±0.04	0.67 ± 0.03	0.16 ± 0.01	0.24 ± 0.00	0.31±0.02	0.30 ± 0.01	0.09 ± 0.00	0.16 ± 0.01	0.23±0.01	0.21 ± 0.01
Maximum Matching Ratio	0.65 ± 0.02	0.74 ± 0.02	0.77±0.04	0.73 ± 0.02	0.25 ± 0.01	0.33 ± 0.01	0.37±0.02	0.34 ± 0.01	0.14 ± 0.00	0.23 ± 0.01	0.29±0.01	0.25 ± 0.01

The table shows the mean and standard deviation values of all metrics. In blue, the metrics for the threshold with the best performance are highlighted.

### 3.3 Execution time and computation complexity

**Figure 9:**
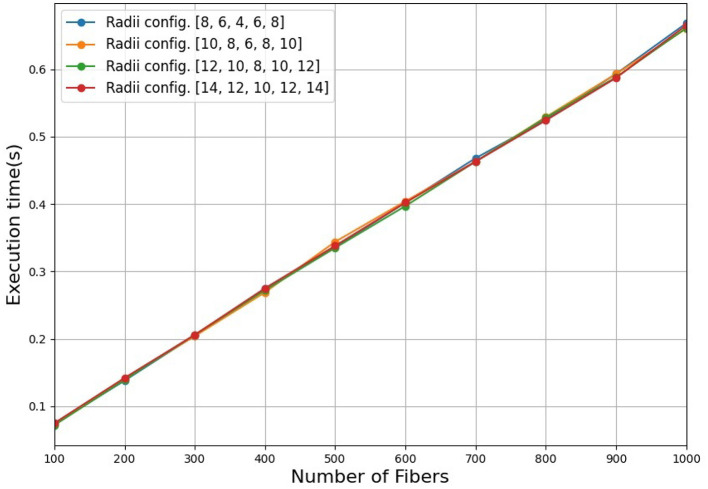
Execution time for the simulation of a bundle containing an increasing number of fibers between 100 and 1,000 fibers using different radii configurations. We used four radii configurations, with higher radii for the external cross-sectional regions (*r*_1_ and *r*_5_), a lower radius for the central region (*r*_3_), and intermediate radii for the regions *r*_2_ and *r*_4_.

The simulator has three steps to simulate a bundle, its time complexity is based on the final steps. The first step is the initialization of the model parameters, which include the centroid of the bundle, the number of fibers, and the radii of the five cross-sectional regions. The algorithm takes a random selection of the radii and number of fibers, from a range of input values. The second step builds the tubular model, which requires the generation of the five circular cross-sectional regions. For each cross-sectional region, the algorithm computes peripheral points within each circle. To establish the eight circular sectors, it employs principles of 3D geometry, including the definition of a plane, the calculation of a vector cross product, and a rotation matrix. This step is independent of the number of fibers and takes constant time. In the third step, for each sector of each region, the algorithm generates uniform random points according to the number of fibers of the bundle. To compute each spline, we take a point within the same sector for each circle as a control point. This step is linear in the number of fibers. Hence, the time complexity for simulating a bundle is O(*N*), where N is the number of fibers of the bundle. Therefore, for simulating a dataset consisting of M bundles the total time complexity is O(*MN*). To experimentally show the time complexity for simulating a bundle, Figure 9 displays the execution time in seconds to simulate a bundle containing an increasing number of fibers between 100 and 1,000 fibers using different radii configurations. As observed, the figure shows a linear-time algorithm to simulate a bundle. In this work, we simulated three datasets with 100, 500, and 1,000 bundles, which were executed in 12.20, 59.84, and 122.68 s, respectively.

## 4 Discussion

We propose a novel bundle simulator algorithm that uses spline curves for fiber representation. Our main purpose is to address the challenge of limited ground truth data for fiber clustering methods. The simulator uses a bundle centroid and the radii of five cross-sectional regions along the centroid to build a tubular model that contains the splines of the simulated bundle. With this approach, we can simulate more realistic bundle shapes than those of the state-of-the-art.

The simulator was tested through the generation of 28 bundles from a DWM bundle atlas. As a result, our method can generate bundles reasonably similar to the atlas bundles, with in general low inter-bundle distances and high intersection percentages between the original and the simulated bundles. As expected, our algorithm performs better for bundles with tubular shapes. It can successfully represent variations in the radii along the bundles. However, sheet-like bundles cannot be represented by our method. This limitation is not as detrimental to the evaluation performed, as the applied fiber clustering algorithms generate tubular clusters. However, it would be very useful to extend the simulator in the future to other bundle shapes, such as sheet-like shapes.

The applicability of the simulator was proven by generating three simulated whole-brain fiber bundle datasets with different numbers of bundles (and fibers) to evaluate the performance of two state-of-the-art fiber clustering algorithms, QuickBundles (QB) and Fast Fiber Clustering (FFClust), for different distance thresholds. We used five metrics to evaluate the quality of the clusters obtained by the algorithms. Thanks to the different metrics used, it was possible to evaluate different aspects of the performance of the algorithms, showing the complexity of evaluating this kind of algorithm. With permutations of the input datasets, it was also possible to evaluate the behavior of the fiber clustering algorithms against permutations of the input data. This yielded interesting results, where both algorithms proved to be robust to the permutations, with a low standard deviation for the five tests. However, the experiment showed that there is no negligible impact of the order of the input data on the results of the two algorithms evaluated. Surprisingly, we found very similar behaviors in both algorithms for the tests and the metrics used, despite the fact that they are very different.

In future work, an important set of existing shapes found in real bundles could be modeled using an extension of the developed framework by delineating non-circular cross-sectional regions. An exception is the corpus callosum if we want to simulate it in a single bundle, since it has a totally different shape that cannot be represented by a linear centroid. However, it should be noted that exploratory algorithms, in general, do not identify it as a single tract.

In any case, we believe that the proposed simulator is an important advance in the state of the art, in order to improve the evaluation of fiber clustering algorithms, since there is no other method with the PhyberSIM features. The method more similar is the proposed by Close et al. (2009), which generates a dataset of numerical phantoms to assess dMRI fiber tracking. This tool uses a collection of numerical constructs known as strands. A strand is defined by a centroid using 3D linear splines, with constant circular cross-sections, meant to represent a collection of axons. The bundles are constructed by a set of strands, fitting scenarios that can be presented during tracking algorithms, such as kissing or crossing bundles. These bundles present almost constant cross-sections and are arranged on a sphere. Also, the bundles and strands do not overlap at their endpoints. On the other hand, our bundle simulator uses a tubular model with different cross-sectional radii, that can represent thickness changes along the bundle. Furthermore, the bundles can overlap at any point and are distributed following a brain shape, leading to more realistic simulated datasets for fiber clustering validation.

Moreover, our simulator can not only be used to validate fiber clustering algorithms, but could also be used to generate other types of simulations. For example, to generate more complex datasets, such as sets of labeled cortical regions and connecting bundles, that would serve as ground truth for the validation of diffusion-based cortical parcellation methods.

## 5 Conclusions

We propose a framework for the simulation of tubular fiber bundles that is easy to use, with a reasonable number of parameters, that can be employed to evaluate fiber clustering algorithms. The experiments performed demonstrated the applicability of the simulator and the metrics used. Despite its limitations, the proposed simulator bridges a gap in the lack of ground truth for the validation of these algorithms. We believe that this tool could be used by the scientific community to test and improve their fiber clustering algorithms. Furthermore, it could be used to generate other types of simulated data, to validate different tractography analysis methods, such as diffusion-based cortical parcellation algorithms. For that, we will make available the codes to generate the simulated data and the whole-brain simulated datasets. Finally, it is important to note that, as future work, we plan to generate other forms of fascicles, and also to add different fiber bundle configurations such as kissing and fanning bundles, as well as other parameters such as fiber density.

## Data availability statement

The datasets presented in this study can be found in online repositories. The names of the repository/repositories and accession number(s) can be found at: https://github.com/elidapoo/Brain_bundle_simulator.

## Ethics statement

The studies involving humans were approved by Comité de Protection des Personnes Île-de-France VII CPP100002/CPP100022, France. The studies were conducted in accordance with the local legislation and institutional requirements. Written informed consent for participation was not required from the participants or the participants' legal guardians/next of kin in accordance with the national legislation and institutional requirements.

## Author contributions

EP: Conceptualization, Formal analysis, Investigation, Methodology, Software, Validation, Writing – original draft, Writing – review & editing. J-FM: Resources, Writing – review & editing. CP: Resources, Writing – review & editing. CH: Conceptualization, Formal analysis, Methodology, Supervision, Writing – original draft, Writing – review & editing. PG: Conceptualization, Formal analysis, Funding acquisition, Methodology, Project administration, Supervision, Writing – original draft, Writing – review & editing.
