# Supplementary material for: PhyberSIM: a tool for the generation of ground truth to evaluate brain fiber clustering algorithms
Source: Front Neurosci. 2024 May 30;18:1396518. doi: 10.3389/fnins.2024.1396518 (PMC11169570; doi:10.3389/fnins.2024.1396518)
Supplement: Supplementary file 1 [file Data_Sheet_1.PDF]

Supplementary Material

1 PRELIMINARY MODEL USING ELLIPTICAL CROSS-SECTIONAL REGIONS

To test the practicability of using a tubular model to represent the bundles, we conducted a short analysis of the shape of the regions of the atlas bundles, by modeling them with elliptical cross-sections. To obtain elliptical cross-sectional regions, three essential parameters are required: the major axis radius, the minor axis radius, and the central point of the ellipse. The central points and the planes of the ellipses are the same as those used for the circular sectors. To calculate the radii for each region, we identified the point farthest from the centroid (Pf1) and a second point (Pf2), also distant but in a perpendicular direction to the previous one. Then, to determine the average major and minor radii for each section, we computed the mean distance between all points in the region to Pf1 and Pf2, respectively, and divided them by two (see an example in Figure S1). Figure S2 shows the model obtained for the left uncinate fasciculus, using the elliptical shapes compared to the circular tubular model.

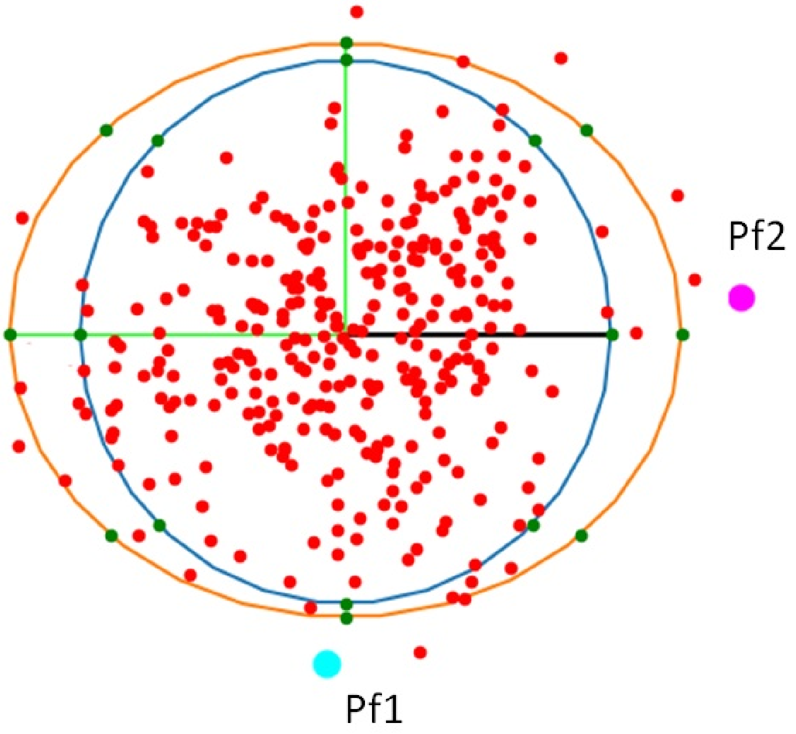

Figure S1. Example of a representation of an intermediate cross-sectional region of the left uncinate fasciculus using the tubular model and the preliminary model based on elliptical cross-sections. The bundle points corresponding to the region are in red. The circular cross-sectional region is in blue, while the elliptical cross-sectional region is in orange. The peripheral points for both regions are in green. The radius of the circular representation is in black, and the major and minor radii of the ellipse are in light green. The farthest point (Pf1) is shown in cyan and the farthest point in a perpendicular direction to Pf1 (Pf2) is in magenta.

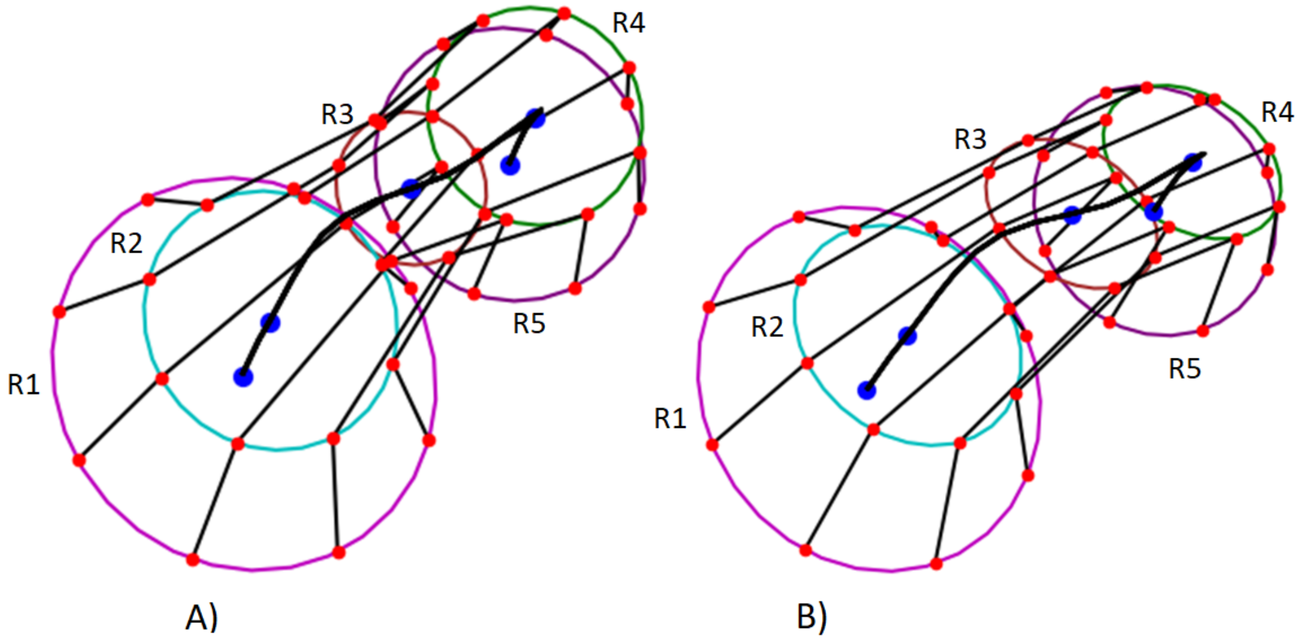

Figure S2. An example of our proposed tubular model (A) with a preliminary model using elliptical cross-sections (B) for the left uncinate fasciculus. The bundle centroid is shown with a thick black line. The end cross-sectional regions appear in magenta (RR=0.9) and purple (RR=0.9), the central cross-sectional region in brown (RR=0.7), and the intermediate cross-sectional regions are in cyan (RR=0.8) and green (RR=0.8). The central points of the cross-sectional regions are in blue, while the peripheral points defining the 8 sectors per region are in red. RR: Elliptical radius ratio.

	Radius Ratio						Proposed simulator		Preliminary model with elliptical cross-sections	
**Atlas Bundles**	**R1**	**R2**	**R3**	**R4**	**R5**	**Mean value**	**ID ± STD (mm)**	**IP(%)**	**ID ± STD (mm)**	**IP(%)**
Anterior Left Arcuate	0.8	1.0	0.7	0.9	1.0	0.9	8.2 ± 1.1	96.4	9.8 ± 1.5	76.7
Anterior Right Arcuate	0.8	1.0	0.7	0.9	1.0	0.9	8.2 ± 1.0	97.9	9.9 ± 1.4	71.2
Left Arcuate	0.8	0.8	1.0	0.6	0.7	0.8	10.5 ± 2.2	44.8	11.4 ± 2.8	33.9
Right Arcuate	0.8	0.8	1.0	0.6	0.7	0.8	10.8 ± 2.2	39.9	11.4 ± 2.6	34.8
Posterior Left Arcuate	0.9	0.9	0.9	0.8	0.8	0.9	6.6 ± 1.0	100	7.0 ± 0.9	100.0
Posterior Right Arcuate	0.9	1.0	0.9	0.8	0.8	0.9	6.7 ± 0.9	100.0	7.1 ± 1.0	100.0
Left Cingulum Fibers	1.0	0.8	0.7	0.6	1.0	0.8	7.9 ± 1.6	86.4	8.6 ± 1.7	78.2
Right Cingulum Fibers	1.0	0.8	0.7	0.6	1.0	0.8	7.2 ± 1.4	89.0	8.6 ± 1.9	78.2
Left Corticospinal Tract	1.0	0.9	0.8	0.9	0.9	0.9	8.1 ± 1.6	87.3	5.8 ± 1.3	100.0
Right Corticospinal Tract	1.0	0.9	0.8	0.9	0.9	0.9	8.0 ± 1.6	87.5	5.9 ± 1.3	99.8
Left Fornix	0.9	0.8	0.7	0.7	0.5	0.7	10.6 ± 1.8	32.9	9.5 ± 1.8	52.9
Right Fornix	0.9	0.8	0.7	0.7	0.5	0.7	10.8 ± 1.7	30.0	9.6 ± 1.9	53.6
Left Inferior Fronto-occcipital	0.8	0.9	0.7	0.9	0.3	0.7	11.9 ± 1.7	13.2	12.8 ± 1.8	11.6
Right Inferior Fronto-occcipital	0.8	0.9	0.7	0.9	0.3	0.7	11.5 ± 1.5	15.5	12.8 ± 1.7	11.2
Left Inferior Longitudinal	1.0	0.7	0.9	1.0	0.9	0.9	10.3 ± 1.8	48.2	11.0 ± 1.8	30.1
Right Inferior Longitudinal	1.0	0.7	0.9	1.0	0.9	0.9	10.2 ± 1.8	49.6	10.9 ± 1.7	30.6
Left Frontal Thalamic Radiations	0.7	0.8	0.9	1.0	0.9	0.8	7.9 ± 1.7	88.5	8.8 ± 1.9	74.5
Right Frontal Thalamic Radiations	0.7	0.8	0.9	1.0	0.9	0.9	8.1 ± 1.8	85.4	9.1 ± 2.1	70.3
Left Superior Motor Thalamic Radiations	0.7	1.0	0.7	0.9	0.8	0.8	7.1 ± 1.1	99.3	7.3 ± 1.6	94.3
Right Superior Motor Thalamic Radiations	0.7	1.0	0.7	0.9	0.8	0.8	7.2 ± 1.3	98.2	7.4 ± 1.6	95.5
Left Occipital Thalamic Radiations	0.7	0.9	0.8	0.8	0.8	0.8	7.6 ± 1.6	90.4	7.6 ± 1.5	96.4
Right Occipital Thalamic Radiations	0.7	0.9	0.8	0.8	0.8	0.8	7.7 ± 1.5	92.8	7.8 ± 1.5	96.8
Left Superior Parietal Thalamic Radiations	0.9	0.9	0.6	0.9	0.9	0.9	6.3 ± 1.1	100.0	6.4 ± 1.0	100.0
Right Superior Parietal Thalamic Radiations	0.9	0.9	0.6	0.9	0.9	0.8	7.5 ± 1.2	100.0	6.3 ± 1.0	100.0
Left Temporal Thalamic Radiations	0.6	1.0	0.7	0.6	0.9	0.8	7.2 ± 1.6	93.8	9.2 ± 2.1	61.5
Right Temporal Thalamic Radiations	0.6	0.9	0.7	0.6	0.9	0.8	7.2 ± 1.4	95.4	9.4 ± 2.0	63.1
Left Uncinate	0.9	0.8	0.7	0.8	0.9	0.8	8.4 ± 1.2	90.5	8.1 ± 1.4	91.4
Right Uncinate	0.9	0.8	0.7	0.8	0.9	0.8	8.6 ± 1.2	88.6	7.9 ± 1.4	91.7

Table S1. Radius ratio for the five elliptical cross-sectional regions (R1-R5) and average radius ratio for the 28 analyzed DWM atlas bundles. Also, results for Intersection percentage (%) (IP) and Inter-bundle Distance ± Standard deviation (mm) (ID ± STD) are shown for our proposed method compared to the preliminary model using elliptical cross-sections. In blue, the eight bundles with improvements are highlighted.

2 SIMULATING BUNDLES OF A DEEP WHITE MATTER BUNDLE ATLAS

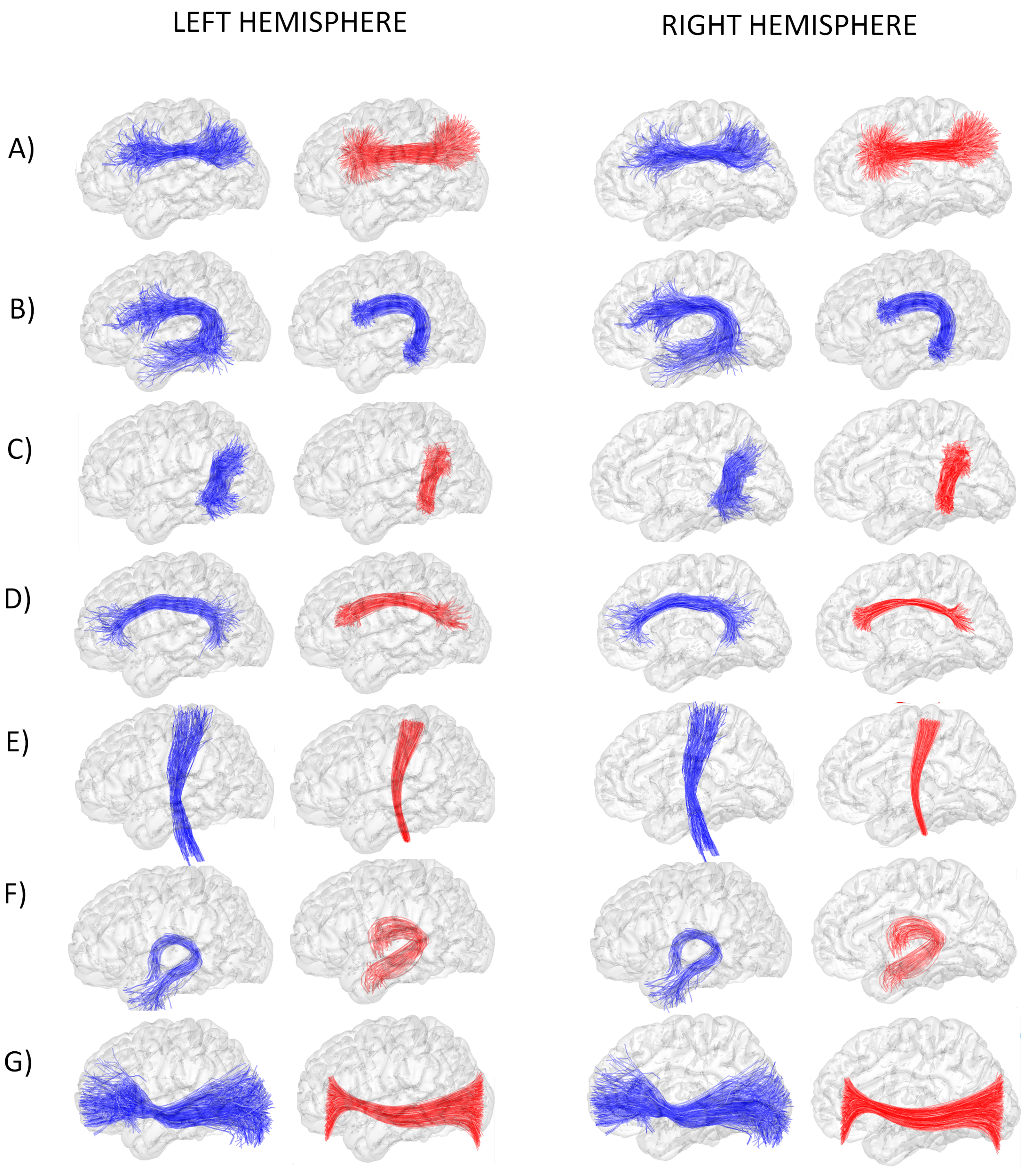

Figure S3. Simulated bundles of the DWM bundle atlas (Guevara et al., 2012) for the left hemisphere (first column) and right hemisphere (second column). Original bundles of the atlas are displayed on the left (blue), and their corresponding simulated bundles are shown on the right in red. A) the anterior arcuate, B) the arcuate, C) the posterior arcuate, D) the cingulum fibers, E) the corticospinal tract, F) the Fornix, G) the Inferior fronto-occipital fasciculus.

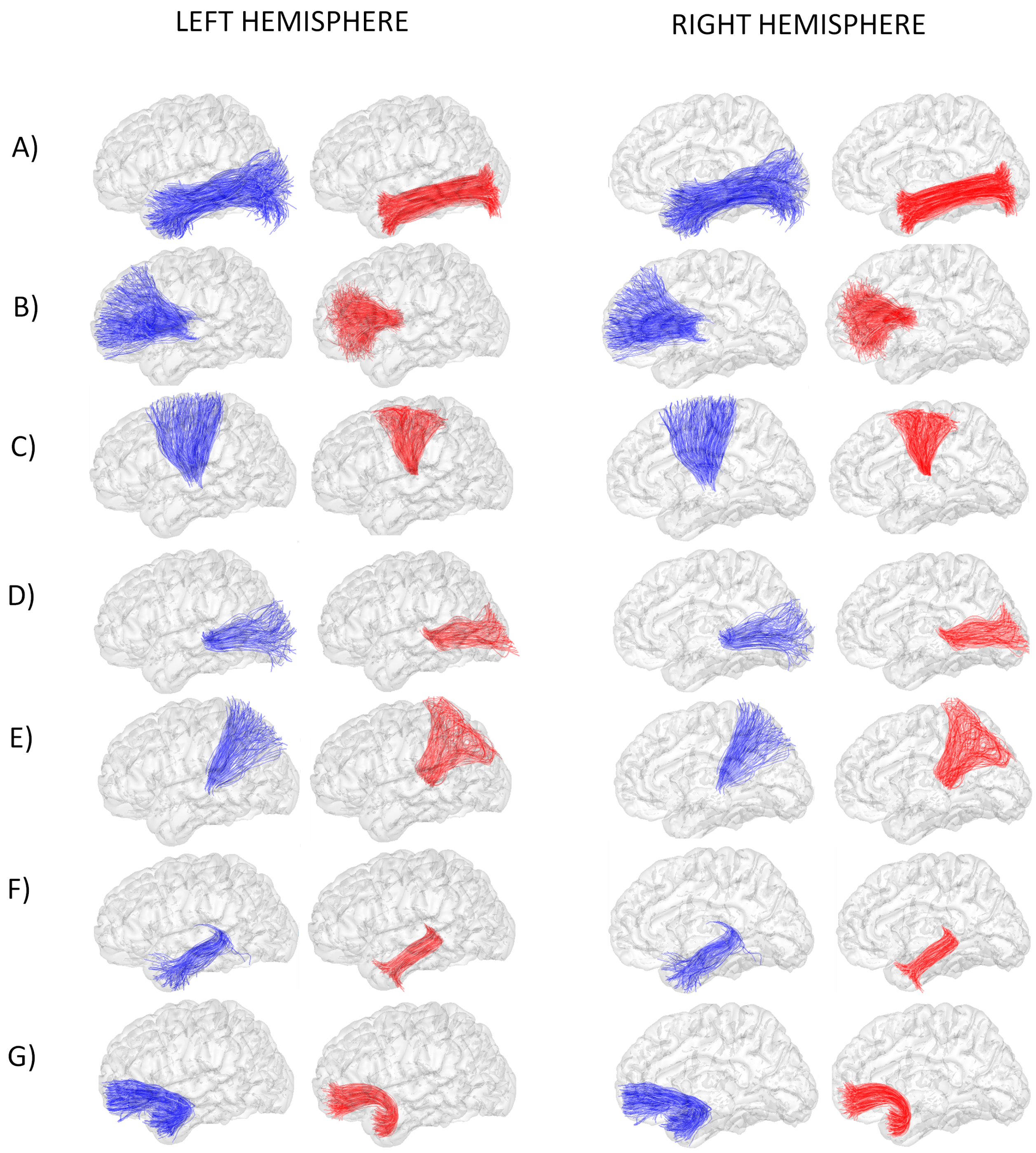

Figure S4. Simulated bundles of the DWM bundle atlas (Guevara et al., 2012) for the left hemisphere (first column) and right hemisphere (second column). Original bundles of the atlas are displayed on the left (blue), and their corresponding simulated bundles are shown on the right in red. A) the iInferior longitudinal, B) the frontal thalamic radiations, C) the superior motor thalamic radiations, D) the occipital thalamic radiations, E) the superior parietal thalamic radiations, F) the temporal thalamic radiations, G) the uncinate fasciculus.

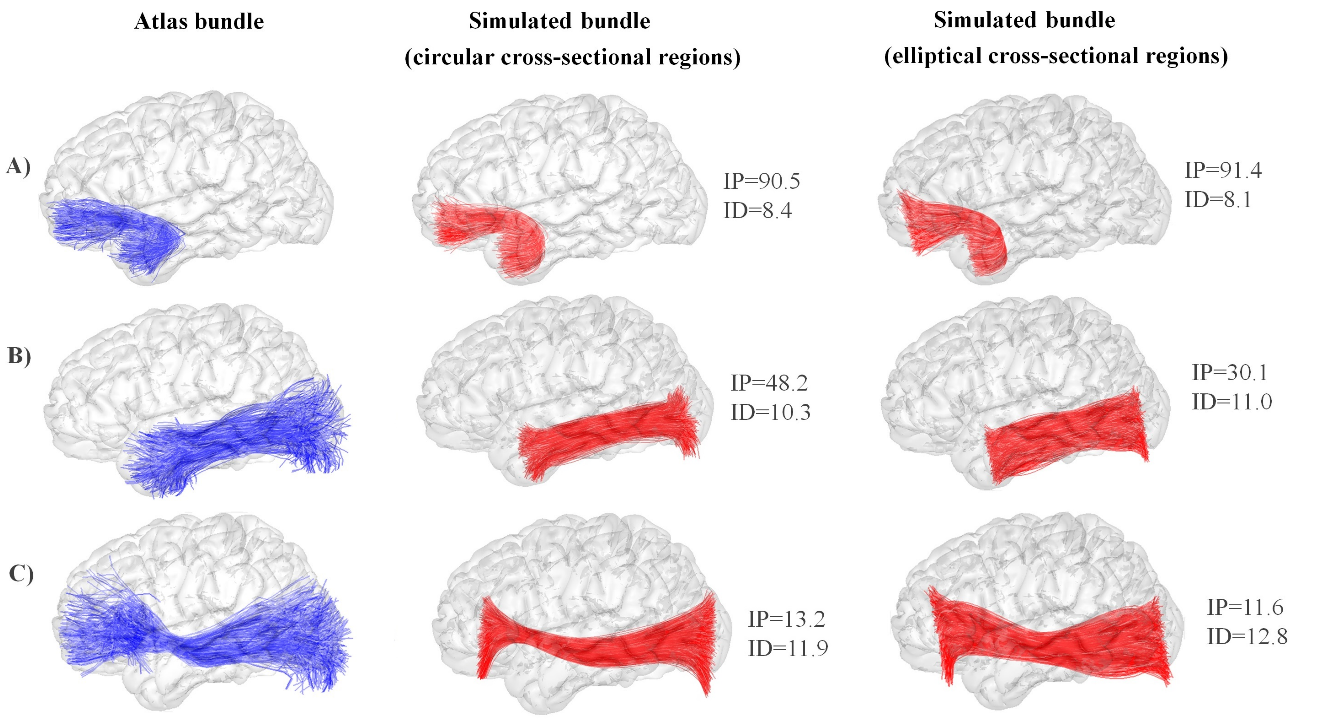

Figure S5. Examples of bundle simulation based on the DWM bundle atlas (Guevara et al., 2012). Original bundles of the atlas are displayed on the left (blue), and their corresponding simulated bundles are shown on the right in red, for the tubular model and the preliminary model using elliptical cross-sections. A) the left uncinate fasciculus, B) the left inferior longitudinal, and C) the Inferior fronto-occipital. IP: Intersection Percentage between bundles (%) ID: Inter-bundle Distance (mm).

3 SIMULATING WHOLE-BRAIN DATASETS

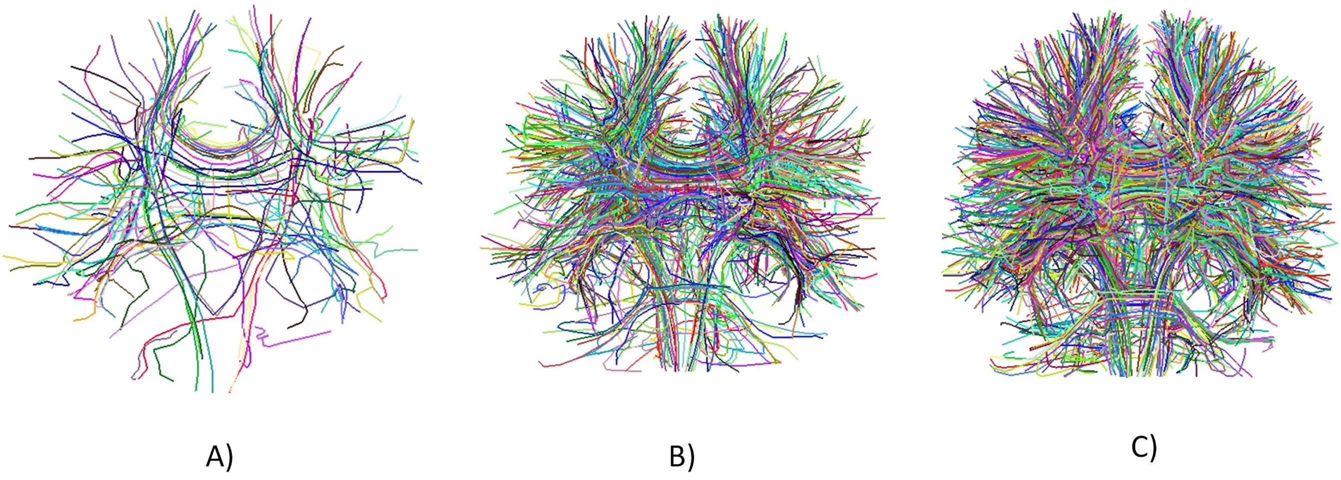

Figure S6. Centroids used for the simulation of the three ground truth datasets of 100 (A) ,500 (B), and 1000 (C) bundles.

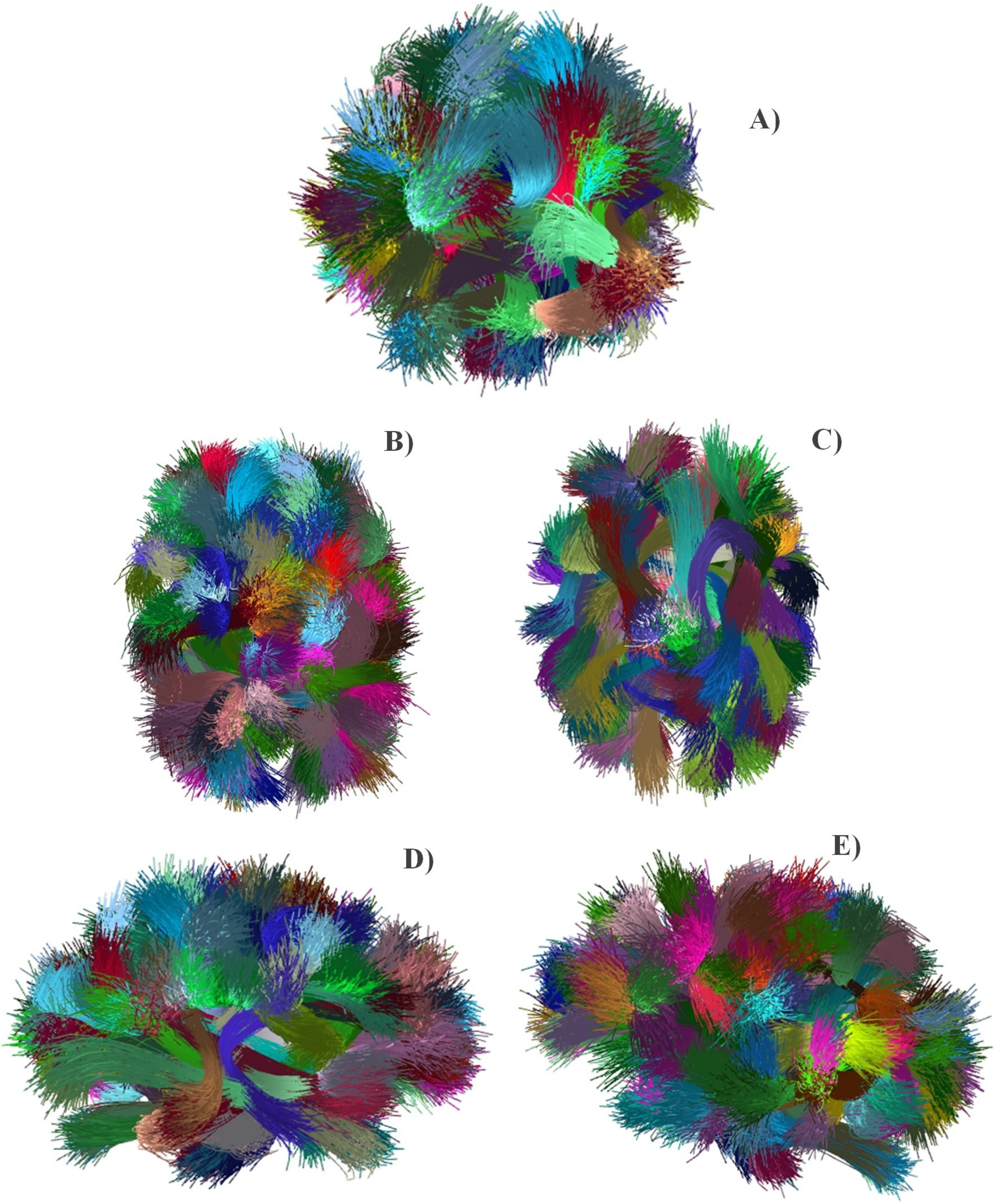

Figure S7. Different views of the ground truth dataset of 100 bundles: A) coronal view, B) superior view, C) inferior view, D) lateral left view E) lateral right view.

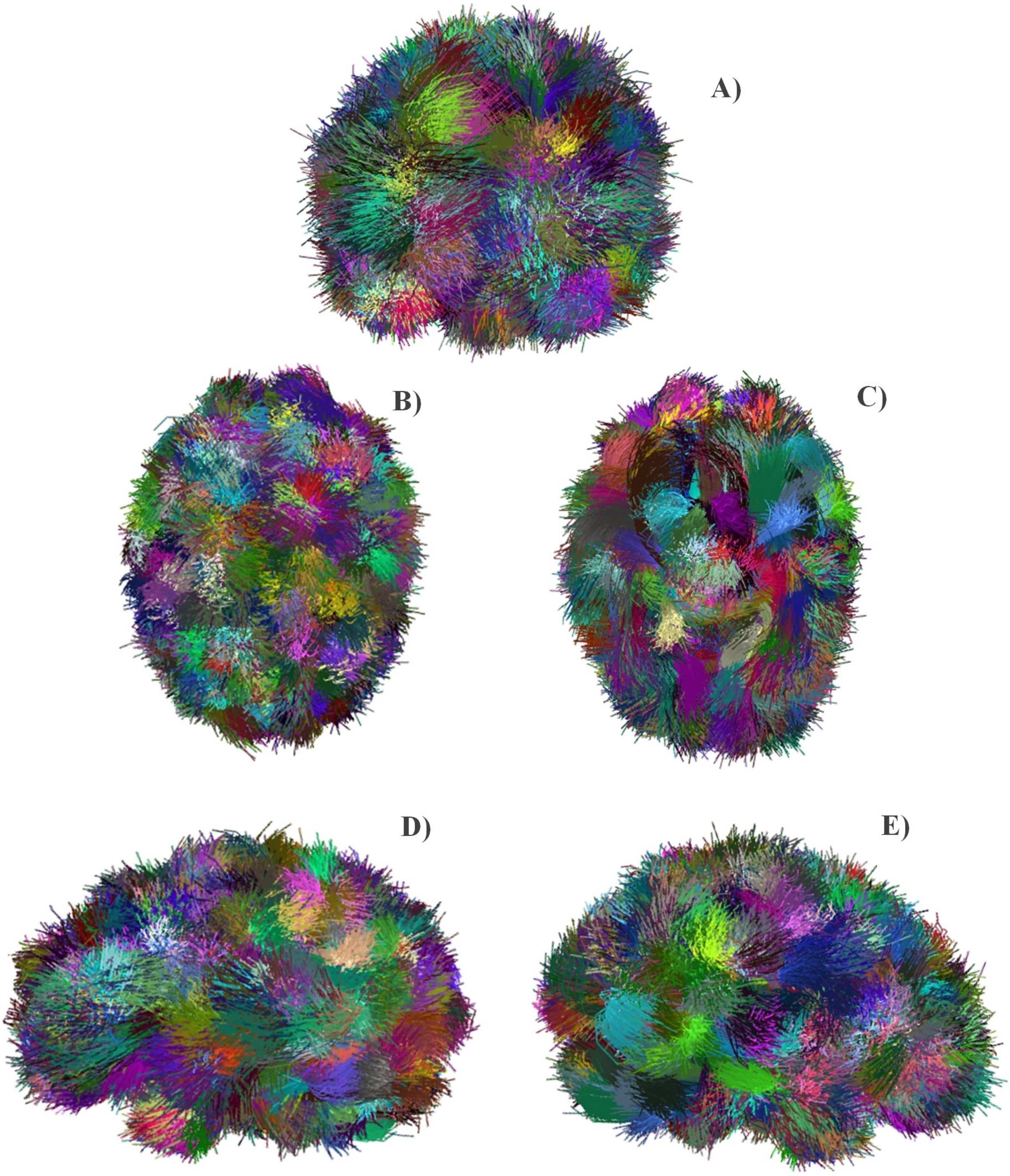

Figure S8. Different views of the ground truth dataset of 500 bundles: A) coronal view, B) superior view, C) inferior view, D) lateral left view and E) lateral right view

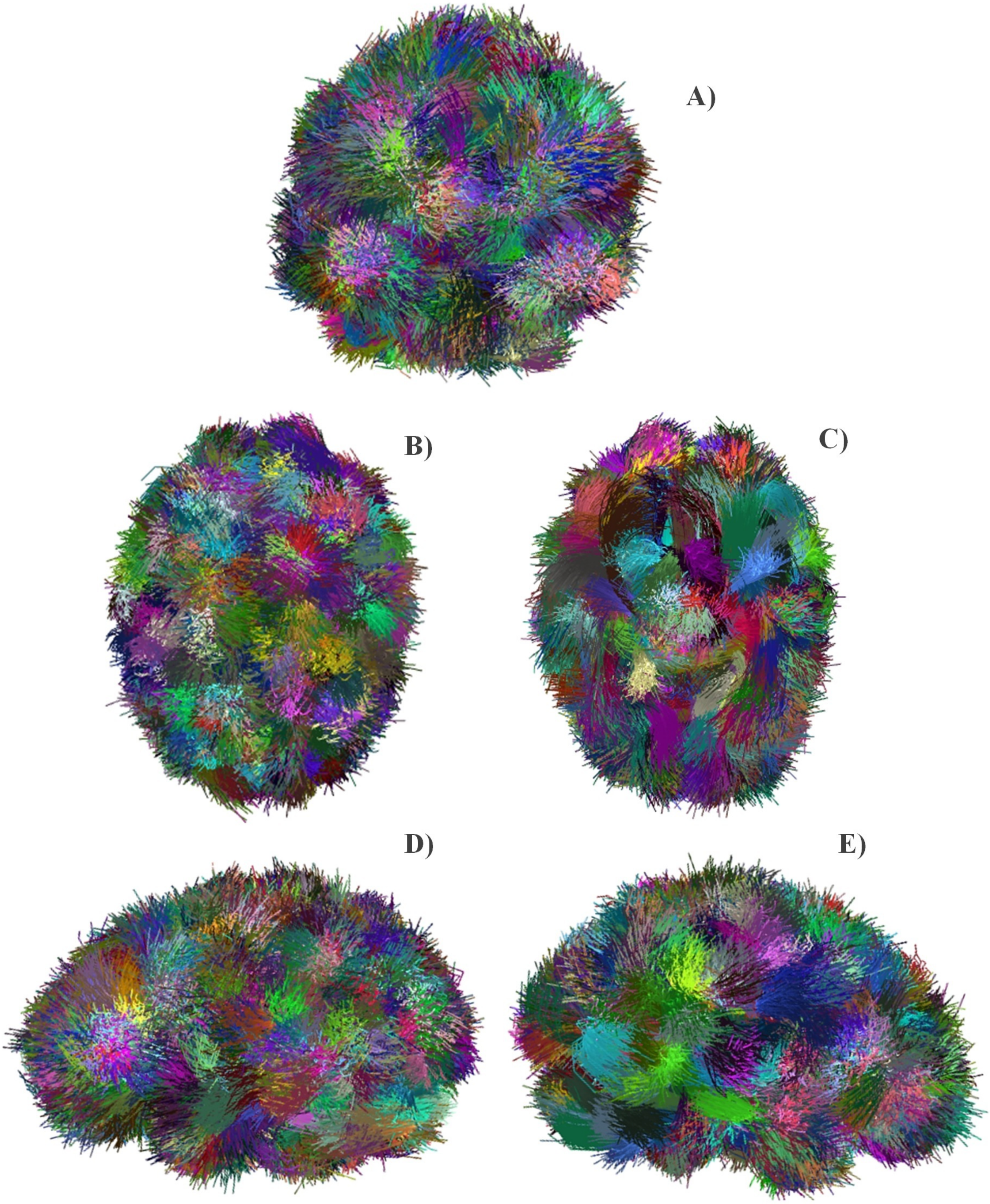

Figure S9. Different views of the ground truth dataset of 1000 bundles: A) coronal view, B) superior view, C) inferior view, D) lateral left view and E) lateral right view

4 COMPUTING FFCLUST PARAMETERS

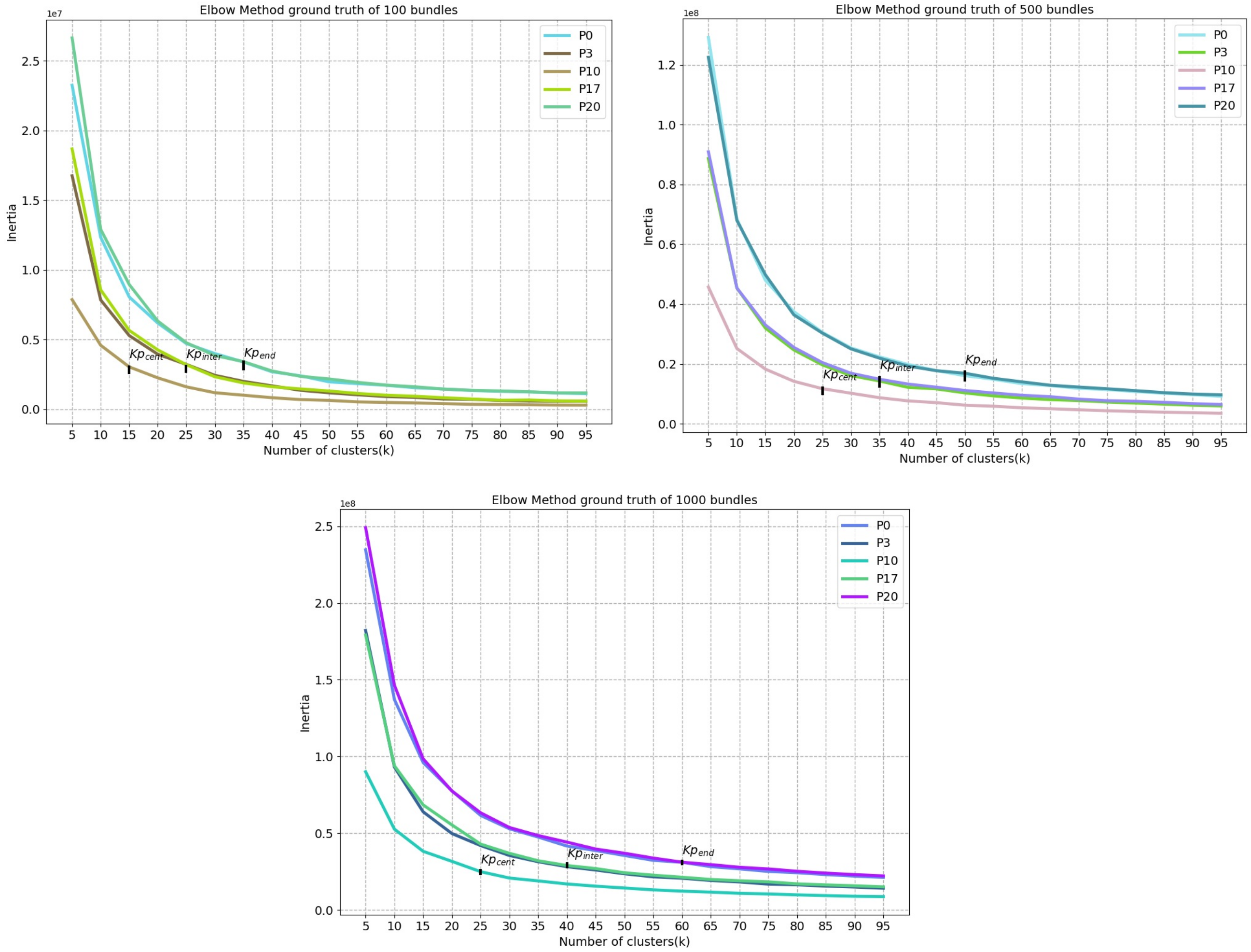

Figure S10. Curves of the Elbow method to compute the number of clusters for each fiber point (0,3,10,17,20) for the MiniBatch K-Means (first step of FFClust). The points selected for the different ground truth datasets are: 100 bundles: $Kp_{end} = 35$, $Kp_{inter} = 25$, $Kp_{cent} = 15$, 500 bundles: $Kp_{end} = 50$, $Kp_{inter} = 35$, $Kp_{cent} = 25$, and 1000 bundles: $Kp_{end} = 60$, $Kp_{inter} = 40$, $Kp_{cent} = 25$.

5 PERFORMANCE METRICS FOR QUICKBUNDLES, FFCLUST AND QBX CLUSTERING ALGORITHMS

**Original clusters**	**100**				**500**				**1000**			
**Thresholds**	**10**	**12**	**15**	**20**	**10**	**12**	**15**	**20**	**10**	**12**	**15**	**20**
True Positives	51.00	79.00	77.00	57.00	156.00	242.00	179.00	71.00	293.00	420.00	276.00	84.00
False Negatives	49.00	21.00	23.00	43.00	344.00	258.00	321.00	429.00	707.00	580.00	724.00	916.00
False Positives	116.00	31.00	14.00	19.00	582.00	226.00	149.00	128.00	1120.00	464.00	296.00	199.00
Clustering-wise Sensitivity	0.78	0.94	0.97	0.98	0.74	0.89	0.94	0.93	0.72	0.86	0.89	0.90
Positive Predictive Value	0.98	0.96	0.90	0.78	0.89	0.83	0.70	0.47	0.85	0.77	0.60	0.34

Table S2. Values of the metrics True Positives (TP), False Negatives (FN), False Positives(FP), Clustering-wise sensitivity (Sn) and Positive Predictive Value (PPV). These were used to obtain the main metrics Precision, Recall and Geometric Accuracy (Acc), needed to assess the performance of the QB algorithm for the three simulated tractography datasets. In blue, the metrics for the threshold with the best performance are highlighted.

**Original clusters**	**100**				**500**				**1000**			
**Thresholds**	**10**	**12**	**15**	**20**	**10**	**12**	**15**	**20**	**10**	**12**	**15**	**20**
True Positives	76.00	79.00	83.00	78.00	133.00	177.00	205.00	172.00	164.00	252.00	316.00	270.00
False Negatives	24.00	21.00	17.00	22.00	367.00	323.00	295.00	328.00	836.00	748.00	684.00	730.00
False Positives	100.00	76.00	43.00	39.00	1060.00	801.00	592.00	516.00	2558.00	1919.00	1403.00	1257.00
Clustering-wise Sensitivity	0.90	0.91	0.94	0.94	0.70	0.76	0.79	0.81	0.64	0.70	0.75	0.76
Positive Predictive Value	0.97	0.97	0.96	0.92	0.89	0.88	0.86	0.81	0.85	0.83	0.82	0.76

Table S3. Values of the metrics True Positived (TP), False Negatives (FN), False Positives(FP), Clustering-wise sensitivity (Sn) and Positive Predictive Value (PPV). These were used to obtain the main metrics Precision, Recall and Geometric Accuracy (Acc), needed to assess the performance of the FFClust algorithm for the three simulated tractography datasets. In blue, the metrics for the threshold with the best performance are highlighted.

**Original clusters**	**100**				**500**				**1000**			
**Thresholds (mm)**	**10**	**12**	**15**	**20**	**10**	**12**	**15**	**20**	**10**	**12**	**15**	**20**
Algorithm clusters	218	146	117	76	1210	761	486	199	2754	1726	943	283
Accuracy	0.86	0.93	0.93	0,87	0.78	0.84	0.80	0.66	0.76	0.79	0.73	0.56
Precision	0.23	0.55	0.70	0.75	0.12	0.30	0.37	0.36	0.09	0.21	0.27	0.30
Recall	0.47	0.74	0.77	0.57	0.25	0.41	0.34	0.14	0.23	0.33	0.24	0.08
F-Measure	0.31	0.63	0.74	0.65	0.16	0.35	0.36	0.20	0.13	0.26	0.25	0.13
Maximum Matching Ratio	0.46	0.72	0.76	0.56	0.24	0.40	0.33	0.14	0.21	0.31	0.23	0.08

Table S4. Metrics values to evaluate the performance of QBX algorithm for the three simulated tractography datasets (100, 500, and 1000 bundles). In blue, the metrics for the threshold with the best performance are highlighted.

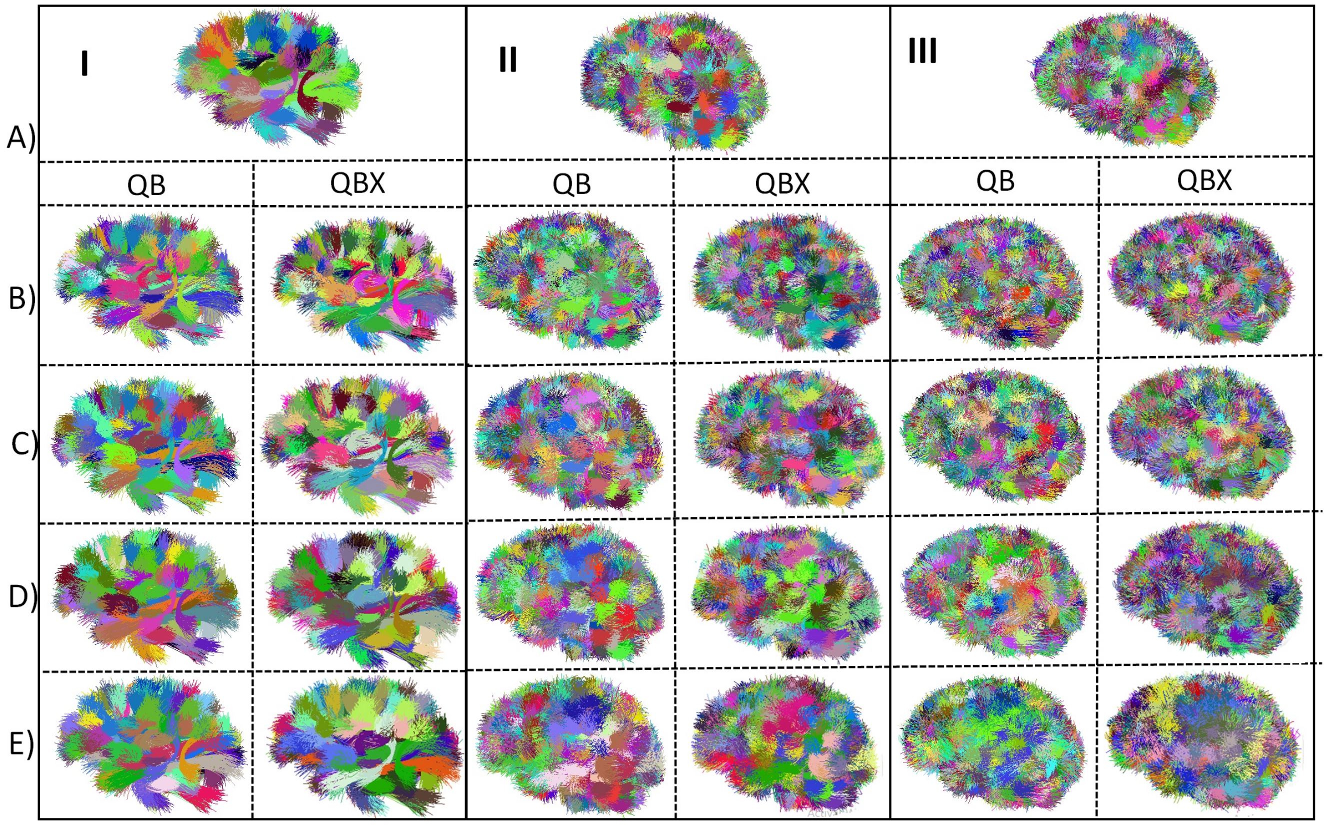

Figure S11. QB and QBX fiber clustering results obtained for simulated tractography datasets with 100 bundles (I), 500 bundles (II) and 1000 bundles (III): A) Original simulated bundles. Clusters obtained by QB and QBX for different thresholds: B) 10 mm, C) 12 mm, D) 15 mm, E) 20 mm.

Original clusters	100				500				1000			
**Thresholds**	**10**	**12**	**15**	**20**	**10**	**12**	**15**	**20**	**10**	**12**	**15**	**20**
True Positives	46.33 ± 6.43	76.00 ± 2.83	74.67 ± 4.04	47.67 ± 4.04	156.00 ± 8.19	223.33 ± 2.52	161.67 ± 6.11	55.00 ± 4.58	297.67 ± 9.71	381.00 ± 13.11	227.00 ± 7.94	62.33 ± 3.51
False Negatives	53.67 ± 6.43	24.00 ± 2.83	25.33 ± 4.04	52.33 ± 4.04	344.00 ± 8.19	276.67 ± 2.52	338.33 ± 6.11	445.00 ± 4.58	702.33 ± 9.71	619.00 ± 13.11	773.00 ± 7.94	937.67 ± 3.51
False Positives	120.67 ± 10.26	38.00 ± 1.41	15.00 ± 3.61	23.00 ± 2.65	582.67 ± 12.58	241.33 ± 2.89	149.33 ± 6.35	127.00 ± 2.00	1113.33 ± 21.94	480.33 ± 20.55	301.00 ± 8.54	200.00 ± 1.00
Clustering-wise Sensitivity	0.77 ± 0.03	0.93 ± 0.00	0.98 ± 0.00	0.98 ± 0.00	0.72 ± 0.01	0.88 ± 0.01	0.93 ± 0.01	0.94 ± 0.01	0.72 ± 0.01	0.85 ± 0.00	0.89 ± 0.01	0.90 ± 0.00
Positive Predictive Value	0.98 ± 0.00	0.95 ± 0.01	0.88 ± 0.01	0.72 ± 0.03	0.87 ± 0.00	0.80 ± 0.00	0.66 ± 0.01	0.43 ± 0.01	0.82 ± 0.00	0.74 ± 0.00	0.55 ± 0.00	0.32 ± 0.01

Table S5. Values variation of the metrics True Positives (TP), False Negatives (FN), False Positives (FP), Clustering-wise sensitivity (Sn) and Positive Predictive Value (PPV) under permutation for the QB algorithm and the three datasets. The table shows the mean and standard deviation of all metrics. In blue, the metrics for the threshold with the best performance are highlighted.

Original clusters	100				500				1000			
**Thresholds**	**10**	**12**	**15**	**20**	**10**	**12**	**15**	**20**	**10**	**12**	**15**	**20**
True Positives	67.33 ± 2.08	76.00 ± 1.73	78.67 ± 4.04	74.67 ± 2.89	136.67 ± 7.23	177.67 ± 3.21	197.00 ± 9.85	176.33 ± 4.73	157.67 ± 1.53	247.00 ± 11.27	309.00 ± 9.17	266.00 ± 8.89
False Negatives	32.67 ± 2.08	24.00 ± 1.73	21.33 ± 4.04	25.33 ± 2.89	363.33 ± 7.23	322.33 ± 3.21	303.00 ± 9.85	323.67 ± 4.73	842.33 ± 1.53	753.00 ± 11.27	691.00 ± 9.17	734.00 ± 8.89
False Positives	121.67 ± 10.97	79.67 ± 5.13	52.33 ± 7.57	46.67 ± 4.16	1049.67 ± 16.20	786.67 ± 2.08	576.33 ± 26.01	496.33 ± 12.74	2492.67 ± 7.37	1903.00 ± 25.24	1414.67 ± 18.61	1229.00 ± 8.72
Clustering-wise Sensitivity	0.88 ± 0.02	0.91 ± 0.01	0.94 ± 0.01	0.94 ± 0.01	0.71 ± 0.00	0.76 ± 0.00	0.81 ± 0.01	0.82 ± 0.01	0.64 ± 0.01	0.70 ± 0.01	0.75 ± 0.01	0.76 ± 0.00
Positive Predictive Value	0.97 ± 0.00	0.97 ± 0.00	0.97 ± 0.00	0.93 ± 0.00	0.88 ± 0.00	0.87 ± 0.01	0.86 ± 0.00	0.81 ± 0.01	0.84 ± 0.00	0.83 ± 0.00	0.81 ± 0.00	0.76 ± 0.01

Table S6. Values variation of the metrics True Positives (TP), False Negatives (FN), False Positives (FP), Clustering-wise sensitivity (Sn) and Positive Predictive Value (PPV) under permutation for the FFClust algorithm and the three datasets. The table shows the mean and standard deviation of all metrics. In blue, the metrics for the threshold with the best performance are highlighted.
